# Supplementary material for: Modifying gut integrity and microbiome in children with severe acute malnutrition using legume-based feeds (MIMBLE): A pilot trial
Source: Cell Rep Med. 2021 May 18;2(5):100280. doi: 10.1016/j.xcrm.2021.100280 (PMC8149470; doi:10.1016/j.xcrm.2021.100280)
Supplement: Document S1. Figure S1 and Tables S1–S5 [file mmc1.pdf]

Cell Reports Medicine, Volume *2*

Supplemental information

Modifying gut integrity and microbiome in children with severe acute malnutrition using legume-based feeds (MIMBLE): A pilot trial

Nuala Calder, Kevin Walsh, Peter Olupot-Olupot, Tonny Ssenyondo, Rita Muhindo, Ayub Mpoya, Jerusa Brignardello, Xuedan Wang, Eleanor McKay, Douglas Morrison, Elaine Holmes, Gary Frost, and Kathryn Maitland

Supplementary Tables 1: Baseline Characteristics

	ConF	InF	CpF	Total
Participants, n	18	20	20	58
Median age in months [Interquartile range)	21 [13-30]	18 [13-23]	18 [14-22]	18 [13-23]
Sex: Male	7 (39)	12 (60)	8 (40)	27 (47)
**Nutritional status and history**				
Median mid-upper arm circumference, cm [IQR]	11.1 [10.4-12.7]	10.5 [10.0-12.0]	11.0 [10.2-11.7]	10.9 [10.2-12.2]
Weight-for-height/length z score <3	10 (56)	13 (65)	12 (60)	35 (60)
Oedema (kwashiorkor)	11 (61)	13 (65)	13 (65)	37 (64)
Oedema severity: severe/generalized	8/11 (72)	10/13 (76)	13/13 (100)	31/37 (84)
Age when feeds introduced (month)	5 [2-6]	3.5 [2.8-6]	5 [2-6]	5 [2-6]
Breast feeding	4/17 (24)	3 (15)	5/19 (26)	12/56 (21)
Previous admission with SAM	1/17 (6)	2/19 (11)	1/19 (5)	4/55 (7)
**Complications at Presentation**				
Diarrhoea	2 (11)	2 (10)	9/19 (47)	13/57 (23)
Pneumonia or Pulmonary Tuberculosis	6 (33)	3 (15)	6/19 (32)	15/57 (26)
Malaria film positive	2/16 (13)	2/17 (12)	0/14 (0)	4/47 (8.5)
HIV Antibody positive	1/15 (7)	1/17 (6)	1/16 (6)	3/48 (6.3)
**Clinical Parameters at Presentation**				
Fever > 37.5°C	4 (22)	8 (40)	6/19 (32)	18/57 (32)
Hypothermia (<36.0 °C)	1 (6)	1 (5)	1/19 (5)	3/57 (5)
Indrawing or deep breathing	1 (6)	2 (10)	3/19 (16)	6/57 (11)
Signs of severe dehydration	0 (0)	1 (5)	6/19 (32)	7/57 (12)
Hyponatraemia (<130 mmol/L)	4/15 (27)	10/18 (55)	11/17 (64)	25/50 (50)
Hypokalaemia (<3.0 mmol/L)	3/16 (19)	11/18 (61)	5/18 (28)	19/52 (37)
Hypoglycaemia (< 3mmol/dl)	2/17 (12)	0/19 (0)	2/18 (11)	4/54 (7)
Severe anaemia (Hb < 5g.dl)	2 (11)	2 (10)	1/19 (5)	5/57 (9)
Lactate > 2 mmols/L	14/17 (82)	15 (75)	10/19 (56)	39/56 (70)
**Risk Score***				
PET score (mean±SD)	1.00 (0.84)	0.95 (0.83)	1.16 (0.69)	1.03 (0.77)

Data are number (%) or median [interquartile range] unless otherwise specified.
% any of capillary refilling time > 2 seconds, temperature gradient or weak pulse
*Risk score: based Paediatric Emergency Triage Score (PET) (George et al 2015)
ConF: Standard recovery feeds F75/F100
InF: Standard recovery feeds with added standard volume of inulin as positive fermentable carbohydrate control
CpF: Standard recovery feeds enriched with cowpea flour
Supplemental Table 1 relates to Figure 1

Supplemental Table 2 Cox proportional hazards model for time to death (up to 28 days)

Variable	Adjusted Hazard Ratio	Lower 95% CI	Upper 95% CI	P
Age (per month)	1.05	0.89	1.02	0.14
Sex (Male vs Female)	1.71	0.47	6.2	0.41
InF versus ConF	1.63	0.33	8.1	0.54
CpF versus ConF	0.66	0.12	3.5	0.63
Paediatric Emergency Triage Score	1.53	0.61	3.8	0.36
Day 1 PYY (per st dev change)	2.8	1.6	5.1	0.0004

Supplemental Table 2 relates to Figure 2

Supplemental Table 3 Summary of biochemical indices and gut microbial phyla arranged by intervention arm and time point, with comparison between time points within intervention groups

	Control Standard Feeds (ConF)				Inulin-enriched Feeds (InF)				Cowpea-enriched Feeds (CpF)			
	ConF1 (n=16)	ConF7 (n=13)	ConF28 (n=13)	Within Group Comparison†	InF1 (n=19)	InF7 (n=13)	InF28 (n=10)	Within Group Comparison†	CpF1 (n=18)	CpF7 (n=15)	CpF28 (n=15)	Within Group Comparison†
Gut hormones (mean (SD))												
PYY pre-feed (pmol/L)	80.5 (93.9)	50.1 (48.6)	25.4 (11.1)	p=0.066	102.1 (83.6)$^{\text{InF28}}$	58.3 (58.8)	26.7 (19.2)$^{\text{InF1}}$	p=0.011	91.8 (74.8)$^{\text{CpF28}}$	55.9 (47.8)	33.8 (16.7)$^{\text{CpF1}}$	p=0.014
PYY post-feed (pmol/L)	82.9 (103.9)	58.4 (72.1)	31.4 (26.7)	p=0.197	98.4 (89.1)$^{\text{InF28}}$	60.2 (68.4)	29.9 (15.0)$^{\text{InF1}}$	p=0.045	65.0 (52.8)	46.7 (34.3)	37.7 (28.0)	p=0.164
GLP-1 pre-feed (pmol/L)	0.8 (0.8)	0.8 (0.3)	1.0 (0.8)	p=0.644	0.7 (0.4)	0.6 (0.4)	0.8 (0.5)	p=0.695	0.8 (0.5)	0.9 (0.7)	1.2 (0.8)	p=0.209
GLP-1 post feed (pmol/L)	0.5 (0.3)$^{\text{ConF7, ConF28}}$	0.8 (0.3)$^{\text{ConF1}}$	0.9 (0.3)$^{\text{ConF1}}$	p=0.006	0.8 (0.5)	0.8 (0.5)	0.9 (0.6)	p=0.887	0.8 (0.4)	0.9 (0.5)	1.0 (0.7)	p=0.701
Faecal Short Chain Fatty Acids (mean (SD))												
Acetate (µmol/L)	1651.0 (770.4)	1091.5 (604.4)$^{\text{ConF28}}$	1853.2 (829.7)$^{\text{ConF7}}$	p=0.021	1413.3 (475.0)	1086.5 (519.0)$^{\text{InF28}}$	1705.4 (673.4)$^{\text{InF7}}$	p=0.035	1542.5 (720.7)	1354.7 (766.8)	1782.4 (768.9)	p=0.287
Butyrate (µmol/L)	843.8 (877.3)$^{\text{ConF7}}$	241.7 (407.3)$^{\text{ConF1, ConF28}}$	1285.3 (816.1)$^{\text{ConF7}}$	p=0.004	753.7 (805.1)$^{\text{InF28}}$	361.5 (538.0)$^{\text{InF28}}$	1470.0 (1102.9)$^{\text{InF1, InF7}}$	p=0.009	760.5 (759.7)	649.4 (638.7)	706.7 (797.7)	p=0.858
Propionate (µmol/L)	1183.3 (1188.8)$^{\text{ConF7}}$	508.1 (419.9)$^{\text{ConF1, ConF28}}$	1697.8 (867.5)$^{\text{ConF7}}$	p=0.003	721.7 (695.8)	517.1 (840.8)	958.2 (736.2)	p=0.375	1229.0 (980.5)	1297.0 (1135.8)	1228.6 (544.1)	p=0.978
Dual Sugar Test (Median (IQR))												
Lactulose (% recovered)	0.22 (0.24)	0.94 (0.99)	0.41 (0.53)	p=0.089	0.30 (0.15)	0.38 (0.55)	0.72 (0.28)	p=0.527	0.13 (0.21)$^{\text{CpF28}}$	0.19 (0.06)	0.36 (0.53)$^{\text{CpF1}}$	p=0.017
Mannitol (% recovered)	1.05 (0.50)	3.15 (5.01)	2.71 (4.98)	p=0.530	0.92 (0.77)$^{\text{InF28}}$	2.27 (0.94)	6.70 (1.93)$^{\text{InF1}}$	p=0.012	0.16 (0.67)$^{\text{CpF28}}$	1.02 (1.77)	2.64 (3.06)$^{\text{CpF1}}$	p=0.038
L:M	0.21 (0.29)	0.36 (0.63)	0.14 (0.10)	p=0.370	0.26 (1.38)	0.35 (0.28)	0.11 (0.18)	p=0.403	0.16 (2.50)	0.17 (0.23)	0.23 (0.16)	p=0.961
Gut microbial species richness and diversity (mean (SD))												
Chao1	53.2 (24.0)	40.8 (12.9)	56.2 (17.0)	p=0.101	54.2 (31.2)$^{\text{InF7}}$	31.1 (13.2)$^{\text{InF1}}$	45.0 (19.7)	p=0.040	54.8 (32.4)	41.6 (21.7)	45.8 (14.8)	p=0.301
Abundance-based Coverage Estimator (ACE)	55.5 (20.9)	42.2 (13.4)$^{\text{ConF28}}$	61.3 (17.5)$^{\text{ConF7}}$	p=0.027	57.2 (32.3)	35.4 (12.7)	61.2 (37.2)	p=0.067	56.0 (31.0)	47.9 (20.6)	57.2 (31.2)	p=0.613
Inverse Simpson Index	4.7 (2.9)	3.2 (1.5)$^{\text{ConF28}}$	6.1 (2.1)$^{\text{ConF7}}$	p=0.011	4.6 (4.0)	2.4 (0.7)	4.6 (1.7)	p=0.084	4.4 (2.3)$^{\text{CpF7}}$	2.8 (1.2)$^{\text{CpF1, CpF28}}$	4.2 (1.6)$^{\text{CpF7}}$	p=0.002
Inverse Shannon Index	1.8 (0.7)	1.4 (0.5)$^{\text{ConF28}}$	2.2 (0.4)$^{\text{ConF7}}$	p=0.006	1.7 (0.7)$^{\text{InF7}}$	1.2 (0.4)$^{\text{InF1, InF28}}$	1.9 (0.5)$^{\text{InF7}}$	p=0.013	1.8 (0.6)	1.3 (0.5)	2.0 (0.4)	p=0.003
Gut Microbial Phyla Relative Abundances (%, mean (SD))*												
Actinobacteria	12.9 (13.1)	15.5 (16)	11.2 (10.6)	BH-FDR p=1.000	11.8 (11.3)	8.6 (9.2)	7.2 (5.3)	BH-FDR p=0.632	11.4 (10.4)	7.2 (8.9)	14.1 (10.8)	BH-FDR p=0.311
Bacteria unclassified	0.2 (0.4)	0 (0)	0 (0)	BH-FDR p=0.731	0.5 (1.5)	0 (0.1)	0.5 (0.8)	BH-FDR p=0.729	0.2 (0.3)	0.1 (0.3)	0.1 (0.1)	BH-FDR p=0.938
Bacteroidetes	16.1 (18.7)	7.8 (5.6)	21.3 (15.8)	BH-FDR p=0.226	8.5 (9.7)	5 (6.7)	23.6 (22.1)	BH-FDR p=0.573	8.1 (9.9)	4.3 (5.4)	9.1 (10.5)	BH-FDR p=0.772

Candidatus Saccharibacteria	0 (0)	0 (0)	0 (0)	BH-FDR p=0.666	0 (0)	0 (0)	0 (0)	BH-FDR p=0.798	0 (0)	0 (0)	0 (0)	BH-FDR p=0.866
Deferribacteres	0 (0)	0 (0)	0 (0)	-	0 (0)	0 (0)	0 (0)	-	0 (0)	0 (0)	0 (0)	BH-FDR p=0.807
Elusimicrobia	0 (0)	0 (0)	0 (0)	-	0 (0)	0 (0)	0 (0.1)	BH-FDR p=0.437	0 (0)	0 (0)	0 (0)	BH-FDR p=1.000
Firmicutes	31.6 (16)	26.9 (16.3)$^{\text{ConF28}}$	47.7 (15.1)$^{\text{ConF7}}$	BH-FDR p=0.038	32.3 (16.8)	25.1 (8.6)	37.4 (15.2)	BH-FDR p=0.503	41.3 (20.2)	31.7 (13.3)	49.9 (15)	BH-FDR p=0.117
Fusobacteria	0.4 (0.7)	0 (0.2)	1.3 (3.9)	BH-FDR p=0.264	0.3 (1.1)	0 (0)	4.1 (12.2)	BH-FDR p=0.418	1.3 (3.1)	0 (0.1)	0.1 (0.2)	BH-FDR p=0.861
Proteobacteria	38.2 (24.3)	49.7 (24.1)	17.8 (14)	BH-FDR p=0.053	45.7 (20)	61.2 (9.5)$^{\text{InF28}}$	27.2 (22.6)$^{\text{InF7}}$	BH-FDR p=0.029	37.3 (20.5)	56.3 (17.4)$^{\text{CpF28}}$	25.4 (18.3)$^{\text{CpF7}}$	BH-FDR p=0.013
Spirochaetes	0 (0)	0 (0)	0 (0)	BH-FDR p=0.824	0.6 (2.4)	0 (0)	0 (0)	BH-FDR p=0.537	0.5 (1.8)	0 (0.1)	0 (0)	BH-FDR p=0.900
Synergistetes	0 (0)	0 (0)	0 (0)	BH-FDR p=0.721	0 (0)	0 (0)	0 (0)	-	0 (0)	0 (0)	0 (0)	-
Tenericutes	0 (0)	0 (0)	0 (0)	BH-FDR p=0.548	0 (0)	0 (0)	0 (0)	BH-FDR p=0.644	0 (0)	0 (0)	0 (0)	BH-FDR p=0.989
Verrucomicrobia	0.7 (2.1)	0.1 (0.2)	0.6 (1.6)	BH-FDR p=0.194	0.4 (1.7)	0 (0)	0 (0.1)	BH-FDR p=0.790	0 (0)	0.4 (1.4)	1.3 (4.5)	BH-FDR p=0.986
Bifidobacterium Genus Relative Abundance (%, mean (SD))												
Bifidobacterium	10.8 (12.5)	13.6 (14.0)	8.9 (10.3)	BH-FDR p=0.894	10.4 (11.4)	8.3 (9.3)	5.7 (5.8)	BH-FDR p=0.850	8.2 (8.2)	5.8 (8.6)	10.9 (8.7)	BH-FDR p=1.000

Abbreviations: PYY: peptide tyrosine tyrosine; GLP-1: glucagon-like peptide 1; L:M: lactulose:mannitol ratio; SD: standard deviation; IQR: interquartile range; BH-FDR p: Benjamini-Hochberg False Discovery Rate Corrected p-value

†Between Group Comparisons report one-way ANOVA (or Kruskal-Wallis one-way ANOVA if non-parametric) p-value with significant ($p<0.05$) pairwise comparisons indicated by superscript determined by Tukey's post-hoc test (or Dunn's post hoc test if Kruskal-Wallis used).

16S rRNA relative abundances compared using multiple groups by Kruskal-Wallis H Test, pairwise Post-Hoc Tukey-Kramer and p-values multiple test correction by Benjamini-Hochberg False Discovery Rate and significant (corrected $p<0.05$) pairwise comparisons indicated by superscript determined by post-hoc Tukey-Kramer

Gut microbial phyla determined by 16S rRNA sequencing; *those with 0% relative abundance and SD were not detected in the 16S rRNA sequencing

$^{\text{ConF1, ConF7, ConF28}}$ denotes post hoc pairwise significant difference from Control Standard Feed Day 1, Control Standard Feed Day 7, Control Standard Feed Day 28 respectively

$^{\text{InF1, InF7, InF28}}$ denotes post hoc pairwise significant difference from Inulin-enriched Feed Day 1, Inulin-enriched Feed Day 7, Inulin-enriched Feed Day 28 respectively

$^{\text{CpF1, CpF7, CpF28}}$ denotes post hoc pairwise significant difference from Cowpea-enriched Feed Day 1, Cowpea-enriched Feed Day 7, Cowpea-enriched Feed Day 28 respectively

Supplemental Table 3 relates to Figure 4

Supplemental Table 4: Summary of biochemical indices and gut microbial phyla arranged by intervention arm and time point, with comparison between intervention groups at each study time point

	Day 1				Day 7				Day 28			
	ConF1 (n=16)	InF1 (n=19)	CpF1 (n=18)	Between Group Comparison‡	ConF7 (n=13)	InF7 (n=13)	CpF7 (n=15)	Between Group Comparison‡	ConF28 (n=13)	InF28 (n=10)	CpF28 (n=15)	Between Group Comparison‡
Gut hormones (mean (SD))												
PYY pre-feed (pmol/L)	80.5 (93.9)	102.1 (83.6)	91.8 (74.8)	p=0.741	50.1 (48.6)	58.3 (58.8)	55.9 (47.8)	p=0.908	25.4 (11.1)	26.7 (19.2)	33.8 (16.7)	p=0.338
PYY post-feed (pmol/L)	82.9 (103.9)	98.4 (89.1)	65.0 (52.8)	p=0.490	58.4 (72.1)	60.2 (68.4)	46.7 (34.3)	p=0.798	31.4 (26.7)	29.9 (15.0)	37.7 (28.0)	p=0.693
GLP-1 pre-feed (pmol/L)	0.8 (0.8)	0.7 (0.4)	0.8 (0.5)	p=0.908	0.8 (0.3)	0.6 (0.4)	0.9 (0.7)	p=0.436	1.0 (0.8)	0.8 (0.5)	1.2 (0.8)	p=0.406
GLP-1 post feed (pmol/L)	0.5 (0.3)^{CpF1}	0.8 (0.5)	0.8 (0.4)^{ConF1}	p=0.041	0.8 (0.3)	0.8 (0.5)	0.9 (0.5)	p=0.724	0.9 (0.3)	0.9 (0.6)	1.0 (0.7)	p=0.851
Faecal Short Chain Fatty Acids (mean (SD))												
Acetate (µmol/L)	1651.0 (770.4)	1413.3 (475.0)	1542.5 (720.7)	p=0.167	1091.5 (604.4)	1086.5 (519.0)	1354.7 (766.8)	p=0.455	1853.2 (829.7)	1705.4 (673.4)	1782.4 (768.9)	p=0.901
Butyrate (µmol/L)	843.8 (877.3)	753.7 (805.1)	760.5 (759.7)	p=0.339	241.7 (407.3)	361.5 (538.0)	649.4 (638.7)	p=0.135	1285.3 (816.1)	1470.0 (1102.9)	706.7 (797.7)	p=0.089
Propionate (µmol/L)	1183.3 (1188.8)	721.7 (695.8)	1229.0 (980.5)	p=0.187	508.1 (419.9)	517.1 (840.8)	1297.0 (1135.8)	p=0.029	1697.8 (867.5)	958.2 (736.2)	1228.6 (544.1)	p=0.053
Dual Sugar Test (Median (IQR))												
Lactulose (% recovered)	0.22 (0.24)	0.30 (0.15)	0.13 (0.21)	p=0.241	0.94 (0.99)^{CpF7}	0.38 (0.55)	0.19 (0.06)^{ConF7}	p=0.017	0.41 (0.53)	0.72 (0.28)	0.36 (0.53)	p=0.712
Mannitol (% recovered)	1.05 (0.50)	0.92 (0.77)	0.16 (0.67)	p=0.194	3.15 (5.01)	2.27 (0.94)	1.02 (1.77)	p=0.311	2.71 (4.98)	6.70 (1.93)	2.64 (3.06)	p=0.158
L:M	0.21 (0.29)	0.26 (1.38)	0.16 (2.50)	p=0.754	0.36 (0.63)	0.35 (0.28)	0.17 (0.23)	p=0.465	0.14 (0.10)	0.11 (0.18)	0.23 (0.16)	p=0.634
Gut microbial species richness and diversity (mean (SD))												
Chao1	53.2 (24.0)	54.2 (31.2)	54.8 (32.4)	p=0.987	40.8 (12.9)	31.1 (13.2)	41.6 (21.7)	p=0.206	56.2 (17.0)	45.0 (19.7)	45.8 (14.8)	p=0.197
Abundance-based Coverage Estimator (ACE)	55.5 (20.9)	57.2 (32.3)	56.0 (31.0)	p=0.984	42.2 (13.4)	35.4 (12.7)	47.9 (20.6)	p=0.142	61.3 (17.5)	61.2 (37.2)	57.2 (31.2)	p=0.917
Inverse Simpson Index	4.7 (2.9)	4.6 (4.0)	4.4 (2.3)	p=0.969	3.2 (1.5)	2.4 (0.7)	2.8 (1.2)	p=0.255	6.1 (2.1)	4.6 (1.7)	4.2 (1.6)	p=0.162
Inverse Shannon Index	1.8 (0.7)	1.7 (0.7)	1.8 (0.6)	p=0.959	1.4 (0.5)	1.2 (0.4)	1.3 (0.5)	p=0.494	2.2 (0.4)	1.9 (0.5)	2.0 (0.4)	p=0.202
Gut Microbial Phyla Relative Abundances (%, mean (SD))*												
Actinobacteria	12.9 (13.1)	11.8 (11.3)	11.4 (10.4)	BH-FDR p=0.953	15.5 (16)	8.6 (9.2)	7.2 (8.9)	BH-FDR p=0.988	11.2 (10.6)	7.2 (5.3)	14.1 (10.8)	BH-FDR p=0.631
Bacteria unclassified	0.2 (0.4)	0.5 (1.5)	0.2 (0.3)	BH-FDR p=0.839	0 (0)	0 (0.1)	0.1 (0.3)	BH-FDR p=0.954	0 (0)	0.5 (0.8)	0.1 (0.1)	BH-FDR p=0.701
Bacteroidetes	16.1 (18.7)	8.5 (9.7)	8.1 (9.9)	BH-FDR p=1.000	7.8 (5.6)	5 (6.7)	4.3 (5.4)	BH-FDR p=1.000	21.3 (15.8)	23.6 (22.1)	9.1 (10.5)	BH-FDR p=1.000

Candidatus Saccharibacteria	0 (0)	0 (0)	0 (0)	BH-FDR p=0.838	0 (0)	0 (0)	0 (0)	BH-FDR p=0.983	0 (0)	0 (0)	0 (0)	BH-FDR p=1.000
Deferribacteres	0 (0)	0 (0)	0 (0)	-	0 (0)	0 (0)	0 (0)	-	0 (0)	0 (0)	0 (0)	-
Elusimicrobia	0 (0)	0 (0)	0 (0)	BH-FDR p=0.983	0 (0)	0 (0)	0 (0)	BH-FDR p=1.000	0 (0)	0 (0.1)	0 (0)	BH-FDR p=0.641
Firmicutes	31.6 (16)	32.3 (16.8)	41.3 (20.2)	BH-FDR p=0.863	26.9 (16.3)	25.1 (8.6)	31.7 (13.3)	BH-FDR p=1.000	47.7 (15.1)	37.4 (15.2)	49.9 (15)	BH-FDR p=0.562
Fusobacteria	0.4 (0.7)	0.3 (1.1)	1.3 (3.1)	BH-FDR p=1.000	0 (0.2)	0 (0)	0 (0.1)	BH-FDR p=1.000	1.3 (3.9)	4.1 (12.2)	0.1 (0.2)	BH-FDR p=0.417
Proteobacteria	38.2 (24.3)	45.7 (20)	37.3 (20.5)	BH-FDR p=0.858	49.7 (24.1)	61.2 (9.5)	56.3 (17.4)	BH-FDR p=0.873	17.8 (14)	27.2 (22.6)	25.4 (18.3)	BH-FDR p=0.821
Spirochaetes	0 (0)	0.6 (2.4)	0.5 (1.8)	BH-FDR p=0.990	0 (0)	0 (0)	0 (0.1)	BH-FDR p=1.000	0 (0)	0 (0)	0 (0)	-
Synergistetes	0 (0)	0 (0)	0 (0)	-	0 (0)	0 (0)	0 (0)	-	0 (0)	0 (0)	0 (0)	-
Tenericutes	0 (0)	0 (0)	0 (0)	BH-FDR p=1.000	0 (0)	0 (0)	0 (0)	BH-FDR p=0.733	0 (0)	0 (0)	0 (0)	BH-FDR p=0.560
Verrucomicrobia	0.7 (2.1)	0.4 (1.7)	0 (0)	BH-FDR p=0.023	0.1 (0.2)	0 (0)	0.4 (1.4)	BH-FDR p=1.000	0.6 (1.6)	0 (0.1)	1.3 (4.5)	BH-FDR p=0.553
Bifidobacterium Genus Relative Abundance (%, mean (SD))												
Bifidobacterium	10.8 (12.5)	10.4 (11.4)	8.2 (8.2)	BH-FDR p=1.000	13.6 (14.0)	8.3 (9.3)	5.8 (8.6)	BH-FDR p=1.000	8.9 (10.3)	5.7 (5.8)	10.9 (8.7)	BH-FDR p=1.000

Abbreviations: PYY: peptide tyrosine tyrosine; GLP-1: glucagon-like peptide 1; L:M: lactulose:mannitol ratio; SD: standard deviation; IQR: interquartile range; BH-FDR p: Benjamini-Hochberg False Discovery Rate Corrected p-value

†Between Group Comparisons report one-way ANOVA (or Kruskal-Wallis one-way ANOVA if non-parametric) p-value with significant (p<0.05) pairwise comparisons indicated by superscript determined by Tukey's post-hoc test (or Dunn's post hoc test if Kruskal-Wallis used).

16S rRNA relative abundances compared using multiple groups by Kruskal-Wallis H Test, pairwise Post-Hoc Tukey-Kramer and p-values multiple test correction by Benjamini-Hochberg False Discovery Rate and significant (corrected p<0.05) pairwise comparisons indicated by superscript determined by post-hoc Tukey-Kramer

Gut microbial phyla determined by 16S rRNA sequencing; *those with 0% relative abundance and SD were not detected in the 16S rRNA sequencing

$^{ConF1, ConF7, ConF28}$ denotes post hoc pairwise significant difference from Control Standard Feed Day 1, Control Standard Feed Day 7, Control Standard Feed Day 28 respectively

$^{InF1, InF7, InF28}$ denotes post hoc pairwise significant difference from Inulin-enriched Feed Day 1, Inulin-enriched Feed Day 7, Inulin-enriched Feed Day 28 respectively

$^{CpF1, CpF7, CpF28}$ denotes post hoc pairwise significant difference from Cowpea-enriched Feed Day 1, Cowpea-enriched Feed Day 7, Cowpea-enriched Feed Day 28 respectively

Supplemental Table 4 relates to Figure 4

Supplemental Table 5 Antibiotic treatments prescribed at any stage from admission to day 28 in each study arm

**Antibiotic, n(%)**	**ConF (n=18)**	**InF (n=20)**	**CpF (n=20)**	**Pearson X^2**	**p-value**
Amoxycillin	1 (5.6)	1 (5.0)	1 (5.0)	0.008*	0.996
Ampicillin	10 (55.6)	14 (70.0)	10 (50.0)	1.750	0.417
Ampiclox	1 (5.6)	3 (15.0)	1 (5.0)	1.493*	0.474
Benzylpenicillin	0 (0)	1 (5.0)	1 (5.0)	1.518*	0.468
Ceftriaxone	12 (66.7)	12 (60.0)	13 (65.0)	0.202	0.904
Chloramphenicol	0 (0)	1 (5.0)	1 (5.0)	1.518*	0.468
Ciprofloxacin	0 (0)	1 (5.0)	0 (0)	2.163*	0.339
Cloxacillin	0 (0)	2 (10.0)	2 (10.0)	3.104*	0.212
Erythromycin	0 (0)	1 (5.0)	0 (0)	2.163*	0.339
Ethambutol	0 (0)	0 (0)	1 (5.0)	2.163*	0.339
Gentamycin	13 (72.2)	17 (85.0)	14 (70.0)	1.496*	0.473
Isoniazid	0 (0)	0 (0)	1 (5.0)	2.163*	0.339
Metronidazole	0 (0)	1 (5.0)	0 (0)	2.163*	0.339
Pyrazinamide	0 (0)	0 (0)	1 (5.0)	2.163*	0.339
Rifampin	0 (0)	0 (0)	1 (5.0)	2.163*	0.339
Cotrimoxazole	0 (0)	0 (0)	1 (5.0)	2.163*	0.339
Number of Different Antibiotics Prescribed					
None	1 (5.6)	2 (10.0)	4 (20.0)	1.979	0.372
One only	3 (16.8)	0 (0)	1 (5.0)	4.950*	0.084
Two	8 (44.4)	5 (25.0)	7 (35.0)	1.589	0.452
Three	6 (33.3)	8 (40.0)	3 (15.0)	3.220	0.200
Four	0 (0)	5 (25.0)	4 (20.0)	**7.553***	**0.023**
*Where the assumptions of the Pearson X^2 are violated, Likelihood Ratio statistic and associated p-value are presented instead.					

Supplemental Table 5 relates to STAR methods Clinical Monitoring section.

Supplemental Figure 1: Study flow diagram

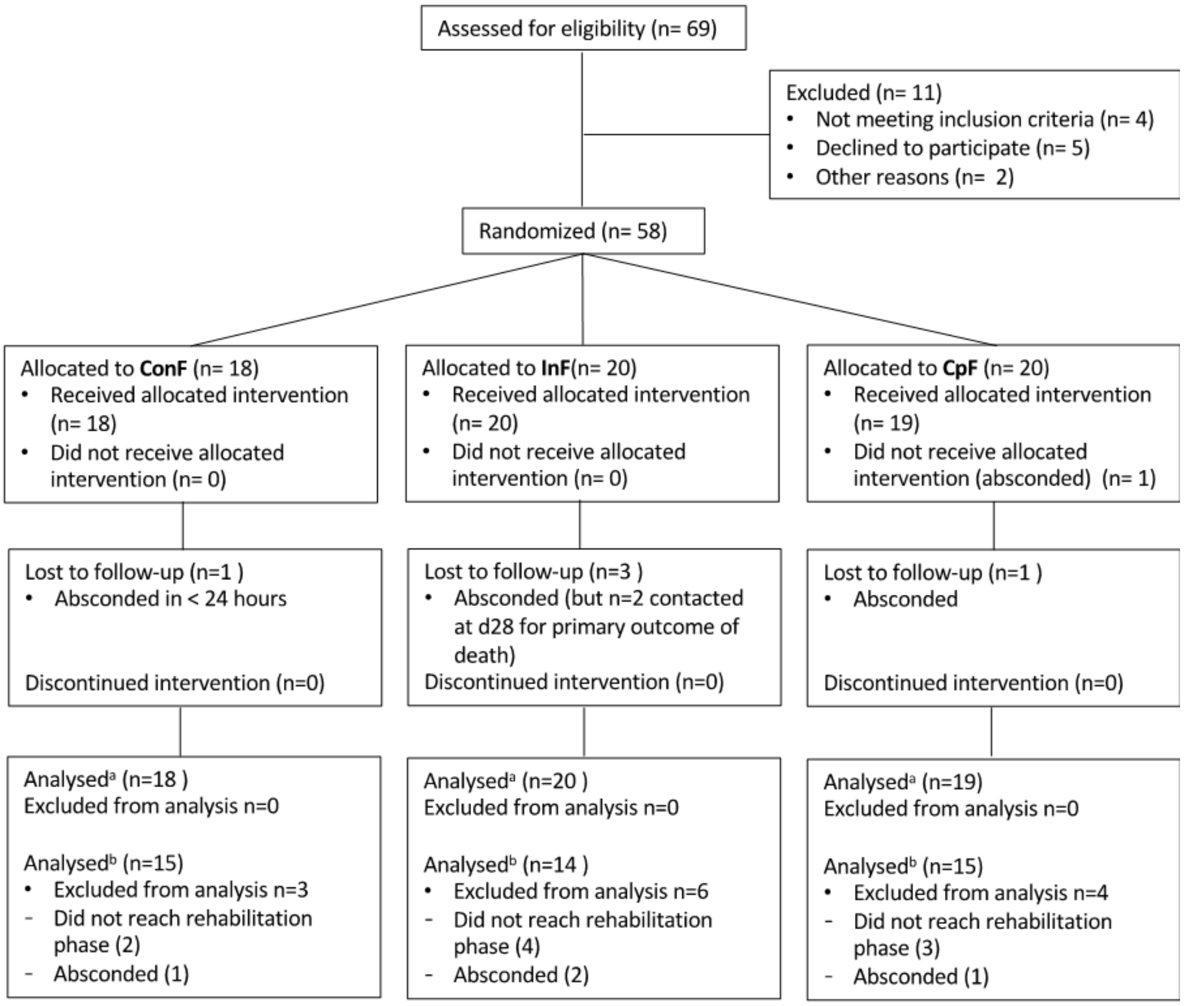

[a] *Analysed with respect to primary outcome survival up to 28 days (time to event analysis)*
[b] *Analysed with respect to primary outcome weight gain > 5g/kg/day (Participants who reached rehabilitation stage)*

Supplemental Figure 1 relates to STAR Methods Experimental Model and Subject Details
