# Supplementary material for: Modifying gut integrity and microbiome in children with severe acute malnutrition using legume-based feeds (MIMBLE): A pilot trial
Source: Cell Rep Med. 2021 May 18;2(5):100280. doi: 10.1016/j.xcrm.2021.100280 (PMC8149470; doi:10.1016/j.xcrm.2021.100280)
Supplement: Document S2. Article plus supplemental information [file mmc2.pdf]

Cell Reports Medicine

Article

Modifying gut integrity and microbiome in children with severe acute malnutrition using legume-based feeds (MIMBLE): A pilot trial

Graphical abstract

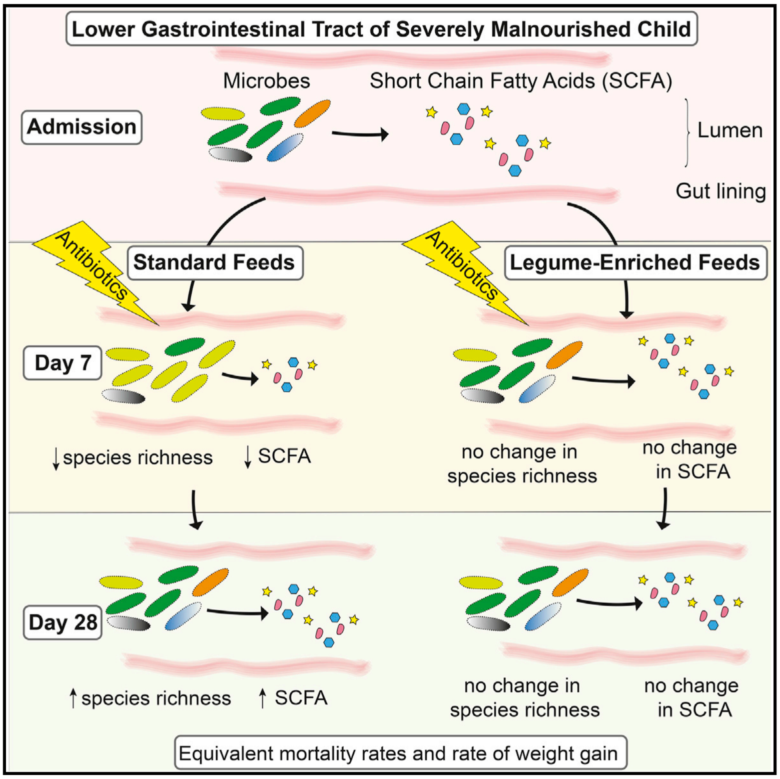

Authors

Nuala Calder, Kevin Walsh, Peter Olupot-Olupot, ..., Elaine Holmes, Gary Frost, Kathryn Maitland

Correspondence

g.frost@imperial.ac.uk (G.F.), k.maitland@imperial.ac.uk (K.M.)

In brief

Calder et al. demonstrate that legume-enriched feeds perform comparably to standard feeds in the treatment of severe acute childhood malnutrition for mortality and weight gain. Fecal bacterial richness and short-chain fatty acid concentration are preserved during antibiotic administration, which has implications for recovery of gastrointestinal health and gut-barrier integrity.

Highlights

- Inpatient feeding with standard or legume-based feeds resulted in similar weight gain
- Legume feeds limited antibiotic-mediated decrease in gut microbiota richness at day 7
- Microbial fermentation was preserved, which has implications for gut health and integrity
- Further testing of legume-enriched feeds in severe acute malnutrition is warranted

Calder et al., 2021, Cell Reports Medicine 2, 100280
May 18, 2021 © 2021 The Author(s).
https://doi.org/10.1016/j.xcrm.2021.100280

CellPress

Cell Reports Medicine

CellPress
OPEN ACCESS

Article

Modifying gut integrity and microbiome in children with severe acute malnutrition using legume-based feeds (MIMBLE): A pilot trial

Nuala Calder,[1,10] Kevin Walsh,[5,10] Peter Olupot-Olupot,[6] Tonny Ssenyondo,[6] Rita Muhindo,[6] Ayub Mpoya,[7] Jerusa Brignardello,[3] Xuedan Wang,[8] Eleanor McKay,[9] Douglas Morrison,[9] Elaine Holmes,[3] Gary Frost,[5,10,*] and Kathryn Maitland[1,2,4,7,10,11,*]

[1]Imperial Centre for Pediatrics and Child Health, Imperial College, St Mary's Campus Norfolk Place, London W2 1PG, UK
[2]Institute of Global Health and Innovation, Imperial College, Faculty Building, South Kensington Campus, Kensington, London SW7 2AZ, UK
[3]Division of Computational and Systems Medicine, Imperial College, Level 2 Faculty Building, South Kensington Campus, Kensington, London SW7 2AZ, UK
[4]Faculty of Medicine, Imperial College, Department of Metabolism, Digestion and Reproduction, Queen Elizabeth the Queen Mother Wing (QEQM) St Mary's Campus, Norfolk Place, London W2 1PG, UK
[5]Division of Diabetes, Endocrinology and Metabolism, Imperial College, 6th Floor Commonwealth Building, Hammersmith Campus, DuCane Road, London W12, UK
[6]Mbale Clinical Research Institute, Busitema University Faculty of Health Sciences, Mbale Campus, Palissa Road, PO Box 1966, Mbale, Uganda
[7]Kenya Medical Research Institute (KEMRI)–Wellcome Trust Research Programme, PO Box 230, Kilifi, Kenya
[8]Department of Food and Nutritional Sciences, The University of Reading, Harry Nursten Building, Pepper Lane, Whiteknights, Reading RG6 6DZ, UK
[9]Stable Isotope Biochemistry Laboratory, Scottish Universities Environmental Research Centre, University of Glasgow, Rankine Avenue, East Kilbride G75 0QF, UK
[10]These authors contributed equally
[11]Lead contact
*Correspondence: g.frost@imperial.ac.uk (G.F.), k.maitland@imperial.ac.uk (K.M.)
https://doi.org/10.1016/j.xcrm.2021.100280

SUMMARY

Case fatality among African children with severe acute malnutrition remains high. We report a 3-arm pilot trial in 58 Ugandan children, comparing feeds targeting disordered gastrointestinal function containing cowpea (CpF, n = 20) or inulin (InF, n = 20) with conventional feeds (ConF, n = 18). Baseline measurements of gut permeability (lactulose:mannitol ratio 1.19 ± SD 2.00), inflammation (fecal calprotectin 539.0 μg/g, interquartile range [IQR] 904.8), and satiety (plasma polypeptide YY 62.6 pmol/l, IQR 110.3) confirm gastrointestinal dysfunction. By day 28, no differences are observable in proportion achieving weight gain >5 g/kg/day (87%, 92%, 86%; p > 0.05), mortality (16%, 30%, 17%; p > 0.05), or edema resolution (83%, 54%, 91%; p > 0.05) among CpF, InF, and ConF. Decreased fecal bacterial richness from day 1 (abundance-based coverage estimator [ACE] 53.2) to day 7 (ACE 40.8) is observed only in ConF (p = 0.025). *Bifidobacterium* relative abundance increases from day 7 (5.8% ± 8.6%) to day 28 (10.9% ± 8.7%) in CpF (corrected p = 1.000). Legume-enriched feeds support aspects of gut function and the microbiome. Trial registration PACTR201805003381361.

INTRODUCTION

In 2016, at least 45% of the 5.6 million child deaths globally were directly or indirectly attributable to undernutrition.[1,2] Severe acute malnutrition (SAM) remains a frequent cause of pediatric hospitalization in much of the developing world,[3] and in Africa, it is associated with high in-hospital mortality rates of ~20%[4,5] and poor long-term outcomes.[6,7] One of the most vulnerable times is early in admission during nutrition rehabilitation, when mortality remains high, with ~70% of in-hospital deaths occurring in the first 7 days[4]. Nutritional (anthropometric) recovery often poorly predicts long-term outcomes,[8] including increased risk of life-threatening events (death and/or re-hospitalization with pneumonia or diarrhea) in the 12 months following initial admission.[7,9]

Factors underpinning the outcome from SAM are complex and multifactorial, ultimately resulting in reduced assimilation of vital nutrients, disruption of the normal gut microbiota, altered gut barrier function, impaired mucosal immunity,[3] and increased risk of gram-negative bacteraemia.[10,11] This has prompted investigation into the role of the gut microbiota,[12,13] gut barrier integrity,[14,15] and microbial translocation[16] in SAM.

© 2021 The Author(s).
This is an open access article under the CC BY license (http://creativecommons.org/licenses/by/4.0/).

Intestinal mucosal integrity and gut microbial diversity can be restored by providing substrates and inducing fermentation in the gastrointestinal tract (GIT).[17,18] Fermentable carbohydrates are increasingly being investigated as potential adjuncts to improve the composition of normal gut microbes and positively influence immunological and metabolic function of the gut.[19,20] Carbohydrate that escapes digestion in the upper GIT (resistant starch and dietary fiber) induces favorable changes in colonic microbiota fermentation.[21] This leads to the generation of short-chain fatty acids (SCFAs), which have a positive influence on gut integrity and nutritional health by improving energy yield, modulation of colonic pH, production of vitamins, and stimulation of gut homeostasis, including anti-pathogenic activities.[22,23] However, it has been demonstrated that in children, SAM is associated with significant relative microbiota immaturity that is only partially ameliorated on standard nutritional rehabilitation feeds given for at least 3 weeks.[13] The study also demonstrated immaturity of microbiota in undernourished children, which directly correlates with the weight-for-height *Z* score. More recent evidence suggests that foods targeted at microbiota recovery that are rich in fermentable carbohydrates have positive effects on linear growth in children with SAM.[24] Part of this diet contained legumes. Legumes, particularly cowpeas, are commonly consumed throughout East Africa. Cowpeas (*Vigna unguiculate*) have been shown to improve the protein digestibility corrected amino acid score (PDCAAS) of traditional African sorghum foods[25] and also have a high resistant starch content.[26] A number of studies have demonstrated that legumes can have a positive effect on the microbiota (e.g., to enhance SCFA production[27] and increase probiotic bacteria[28]). The resistance of starch in cowpeas to small intestinal digestive enzymes is due to a number of factors such as the structure of the starch granule and the entrapment of the starch in the cell.[29]

We hypothesized that the introduction of a legume-enriched feed containing fermentable carbohydrates in children that have complex SAM would provide a safe and cost-effective means of improving outcomes by restoring gut mucosal integrity and enhancing immunity, thereby reducing diarrhea and the risk of systemic infection. To date, studies examining the feasibility and effectiveness of legume-enriched feeds or diets in modulating gut health and microbial composition have been limited to community-based management of chronic or moderate acute malnutrition.[30–32] Our study investigates the use of a legume-enriched feed in the earliest stages of inpatient stabilization in acutely unwell children with SAM at the highest risk of death. Children during this stage of treatment are more likely to require antibiotic treatment,[33] which can negatively impact the gut microbiota and increase risk of diarrhea.[34,35] To provide proof of this principle in Ugandan children with SAM, we assessed the effects on intestinal permeability (urine dual sugar test), SCFA production, gut inflammation (fecal calprotectin), gut microbiome, endocrine measures of satiety (polypeptide YY [PYY] and glucagon-like peptide-1 [GLP-1]), and clinical outcomes in a pilot study in children randomized 1:1:1 to standard nutritional milk feeds with an added source of fermentable carbohydrate: (1) milled cowpea flour (experimental legume-based: cowpea [Cp]-enriched feeds [F] [CpFs]), (2) inulin (In) (experimental non-plant-based: inulin-supplemented feeds [InFs]), or (3) standard nutritional feeds F75 followed by F100 milk (Nutriset, France) (control feed: ConF). Children enrolled on admission to hospital (day 1) were followed up on days 7 and 28 post-enrollment. All other treatments were standardized across the study group.

RESULTS

Feed development, including assessment of the impact of CpFs on fecal microbiota *in vitro* (Figures 1A and 1B) and trial methodology, are presented in the STAR Methods section. Modifying intestinal integrity and microbiome in malnutrition with legume-based feeds (MIMBLE) was a single-center (Mbale Regional Referral Hospital), open-label, proof-of-principle randomized comparator trial evaluating safety and feasibility of three feeding strategies. Trial flow and baseline characteristics are summarized in STAR Methods and Table S1. All three feeds were well tolerated with no reported palatability problems. Mean duration (± SD) of feeding days was 14 ± 8 for ConFs, 14 ± 9 for InFs, and 18 ± 9 for CpFs (Figure 1C). There was no difference in adverse event rates, which were all judged to be causally unrelated to intervention.

Anthropometric recovery and mortality across the cohort

The primary endpoint of nutritional rehabilitation (moderate weight gain > 5 g/kg/day[36]) was achieved in most children (37/42; 88%), with no difference among intervention arms. The co-primary endpoint was 28-day mortality, which, overall, remained high (21%), with no difference among arms (p value for log-rank test: p = 0.48; Figure 1D). Duration of hospital stay did not vary significantly among the intervention arms: ConF median stay was 15.5 days (interquartile range [IQR] 11.0), InF median stay was 10.5 days (IQR 17.0), and CpF median stay 20.0 days (IQR 14.0) (Kruskal-Wallis test, p = 0.183).

Baseline physiological measures and relationship to mortality

Baseline characteristics were relatively similar across the three arms with some imbalances (more diarrhea/dehydration in the CP group). Overall, children that died (versus survivors) had a lower fecal gut microbiota diversity (Figure 2A). We found a significant difference at phylum level, with high levels of *Proteobacteria* in those children that died (relative abundance 52.5%; SD 11.6) compared to those who survived (37.9%; SD 22.8; Benjamini-Hochberg false discovery rate [FDR]-corrected p = 0.04) (Figure 2B). *Proteobacteria* are a large class of bacteria that include a number of pathogens (*Salmonella enterica*, *Pseudomonas xanthomarina*, *Hafania alvei*, *Klebsiella pneumoniae*, and *Escherichia coli*) and that have been shown to be enriched in children with severe undernutrition.[37] In the current study, these species were not identified, and the significant differences existed at the phylum and family levels only. At the family level, there was significantly higher levels of *Enterobacteriaceae* in the stool of children that died (51.9%; SD 10.1) compared to those who survived (36.5%; SD 23.4; Benjamini-Hochberg FDR-corrected p = 0.046) (Figure 2C). Despite a similar microbial diversity, the children that did not recover had significantly lower fecal propionate and butyrate (Figures 2D and 2E), suggesting a

CellPress
OPEN ACCESS

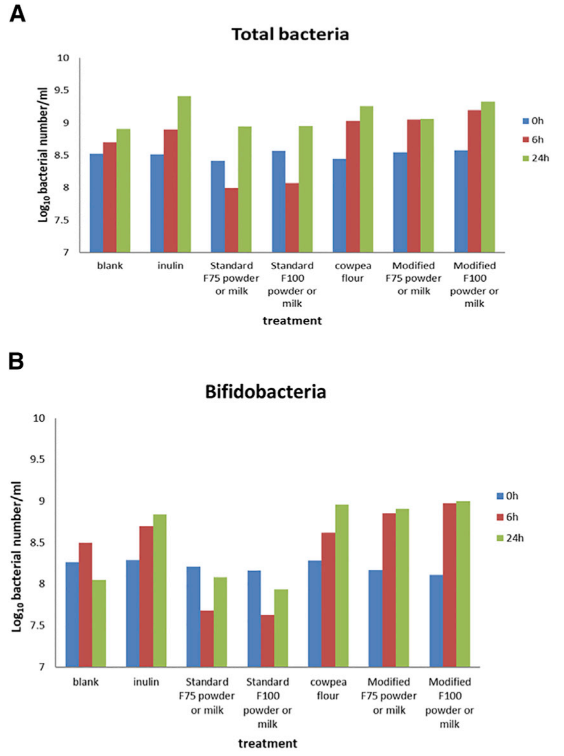

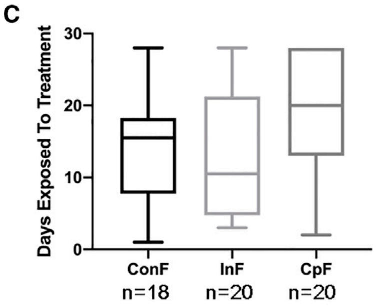

D Primary and secondary outcomes

	ConF	InF	CpF	Total	P*
**Co-primary outcomes**					
**Number achieving weight gain (>5g/kg/day)**	12/14 (86%)	12/13 (92%)	13/15 (87%)	37/42 (88%)	ConF vs InF > 0.99<br>ConF vs CpF > 0.99<br>InF vs ConF > 0.99
**28-Day Mortality**	3/18 (17%)	6/20 (30%)	3/19 (16%)	12/57 (21%)	ConF vs InF = 0.45<br>ConF vs CpF > 0.99<br>InF vs CpF = 0.45
**Secondary Outcomes**					
**Number achieving oedema resolution**	10/11 (91%)	7/13 (54%)	10/12 (83%)	27/36 (75%)	ConF vs InF = 0.08<br>ConF vs CpF > 0.99<br>InF vs CpF = 0.20

ConF: Standard recovery feeds F75/F100
InF: Standard recovery feeds with inulin as positive fermentable carbohydrate control
CpF: Standard recovery feeds enriched with cowpea flour
* P value for Fisher's exact test

Figure 1. Pre-clinical study *in vitro* batch culture: microbiota for the three feeds, days exposed to nutritional treatment, and co-primary endpoint (survival)
(A and B) Total bacteria and *Bifidobacteria* following the batch culture experiment performed in triplicate.
(C) Number of days children received nutritional feeds/treatment by arm (SD). ConF, n = 18; InF, n = 20; CpF, n = 20.
(D) Primary and secondary outcomes; data for weight gain to day 28 are reported for survivors to this time point.
Additional characteristics are summarized in Table S1.

lower functionality of the microbiota. These are two SCFAs that play an important role in colonic health.[38,39] Specifically, butyrate is important as an energy source for colonocytes and for maintaining epithelial integrity.[40] Although all SCFAs cause smooth muscle relaxation and vasodilation, propionate seems to have a specific effect on mesenteric small arteries enhancing blood flow to the colon, which is critically important in maintaining oxygen to a highly metabolic tissue.[41]

When assessed at baseline, the urinary lactose:mannitol (L:M) ratio was significantly higher (p = 0.002) in those children who subsequently died (6.6 IQR [6.4]) within 28 days compared with those children who survived (0.2 IQR [0.5]) (Figure 2H), suggesting that the children who died had impaired mucosal intestinal function and increased gut permeability. These results are similar to those previously reported in a systematic review by Denno and colleagues.[42] Similarly, urinary %L was significantly higher (p = 0.004) in children who subsequently died (0.4 IQR [1.6]) over the 28 days of follow up compared with those children who survived (0.1 IQR [0.3]). There was no difference in urinary % M at baseline between those children who survived and those who died.

We measured the anorectic gut hormone PYY to understand a driver of appetite regulation. The gut hormone PYY was significantly higher at baseline in those that died (Figure 2I), an observation that points to intestinal dysfunction.[43] In a survival analysis, higher baseline PYY was significantly associated with mortality in the whole cohort (Hazard Ratio]HR] = 2.2 [95% confidence interval (CI) 1.4-3.4], p = 0.0005; Figure 2I). In a Cox proportional hazards model containing age, sex, feed intervention, pediatric emergency triage (PET) score, and baseline PYY, only PYY was significantly associated with mortality (adjusted HR = 2.8 [CI 1.6–5.1]; p = 0.0004) (Table S2). Overall, these results demonstrate that the gut is highly dysfunctional in children who die, with potential increases in pathogenic bacteria and low levels of butyrate. The impairment of gut-barrier integrity (increased L:M ratio) and a greater increase in the anorectic hormone PYY suggest a pathophysiological connection between intestinal dysfunction and mortality.

Changes in GIT health over time on standard nutritional feeds

The World Health Organization (WHO) standard feeding group (ConF) offered the opportunity to investigate the impact of the WHO standard nutritional therapy program over a 28-day follow up on GIT health. The fecal microbiota diversity showed very little change over the 28-day intervention or change in major phyla

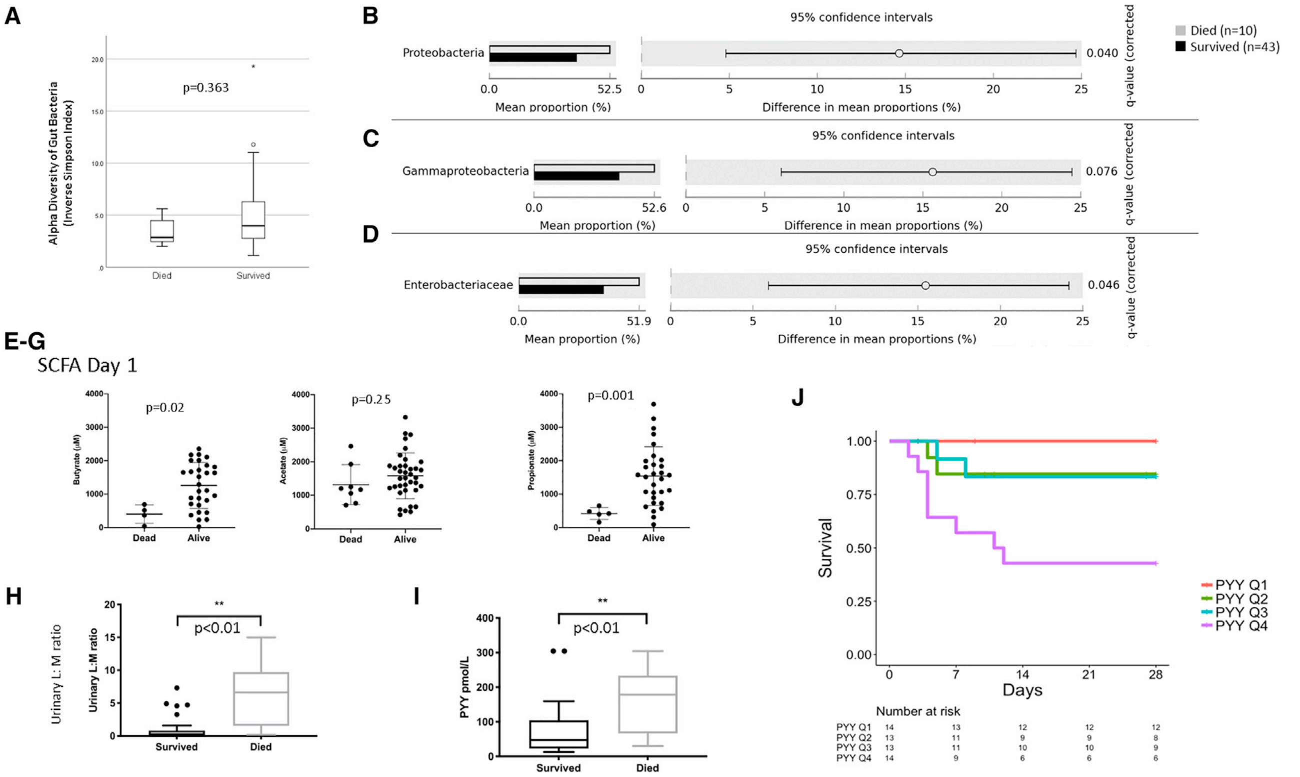

Figure 2. Baseline parameters of children with respect to outcome (death versus survival) for all children irrespective of intervention arm

(A–D) Baseline diversity of the microbiota by outcome (A), phylum (B), class (C), and family level (D). Benjamini-Hochberg FDR-corrected p values are shown. n = 53 (died n = 10/survived n = 43).

(E–G) Fecal SCFAs. (E) n = 33 (n = 4 died, n = 29 survived); (F) n = 45 (n = 8 died, n = 37 survived); (G) n = 35 (n = 5 died, n = 30 survived).

(H) Urinary L:M ratio. n = 37 (n = 7 died, n = 30 survived).

(I) Plasma concentrations of PYY (peptide tyrosine tyrosine) at baseline. n = 54 (n = 12 died, n = 42 survived).

(J) Kaplan-Meier plots for the quartiles of plasma PYY and survival status. n = 54

Results are presented as medians with 95% confidence limits (CLs). Comparisons between intervention arms are made by Kruskal-Wallis one-way analysis of variance and Mann-Whitney U test. Baseline fecal samples were available for 53/58 children only, hence the results reported above for diversity (A), bacterial phyla (B), classes (C), and families (D) and fecal SCFAs are reported for available samples only. Sample numbers vary due to sample availability. Additional Cox proportional hazards models are reported in Table S2.

(Figures 3A and S1). This observation is similar to findings of the Malawian twin study, where there was no change in the microbiota of children with kwashiorkor when exposed to ready-to-use feeds.[44] However, we do show change at the phylum level, with high percentages of *Proteobacteria* on days 1 and 7 compared to day 28 (Figure 3B). This phylum is very diverse but contains the *Gammaproteobacteria* class, which was in particularly high density in children who subsequently died. Enrichment of *Proteobacteria* has previously been reported in children with SAM, more specifically in those with the kwashiorkor phenotype.[37] The reduction in *Firmicutes* and *Bacteroidetes* is of interest, as these two saccharolytic phyla are responsible for SCFA production.[45] Here, we report that fecal SCFA concentrations on admission to hospital were approximately one-third of the concentration of those reported in healthy African infants.[46] There are different sampling and analytical methods used to determine fecal SCFAs in healthy children in this study, allowing for some comparison. Ideally, in the future, it will be necessary to derive normative data using the same method. However, there was a suppression of the SCFAs propionate and butyrate at day 7 (to about one-tenth of the normal concentrations) that recovered by day 28 (Figure 3C). We suspect that the suppression of SCFAs at day 7 may have been due to the use of antibiotics,[47] which recovered once antibiotic treatments were stopped. Antibiotic treatments given in this study are summarized in Table S4; no difference was observed in the types used among the intervention arms.

On day 1, fecal calprotectin was high (median 539 μg/g stool; IQR: 904.75) and similar among the three intervention arms. There was a stepwise decrease in fecal calprotectin over time, when comparing day 1 versus day 7 ($p = 0.049$) and day 1 versus day 28 ($p = 0.0068$, adjusted for multiple comparisons) (Figure 3D). Similar trends were also seen for PYY (Figure 3E), suggesting that GIT is improving in all intervention arms over time.

No significant change was observed in urinary median mannitol excretion, but lactulose excretion increased significantly on day 7 (median: 0.94%; lactulose-recovered IQR: 0.99) compared with day 1 (median: 0.21%; lactulose-recovered

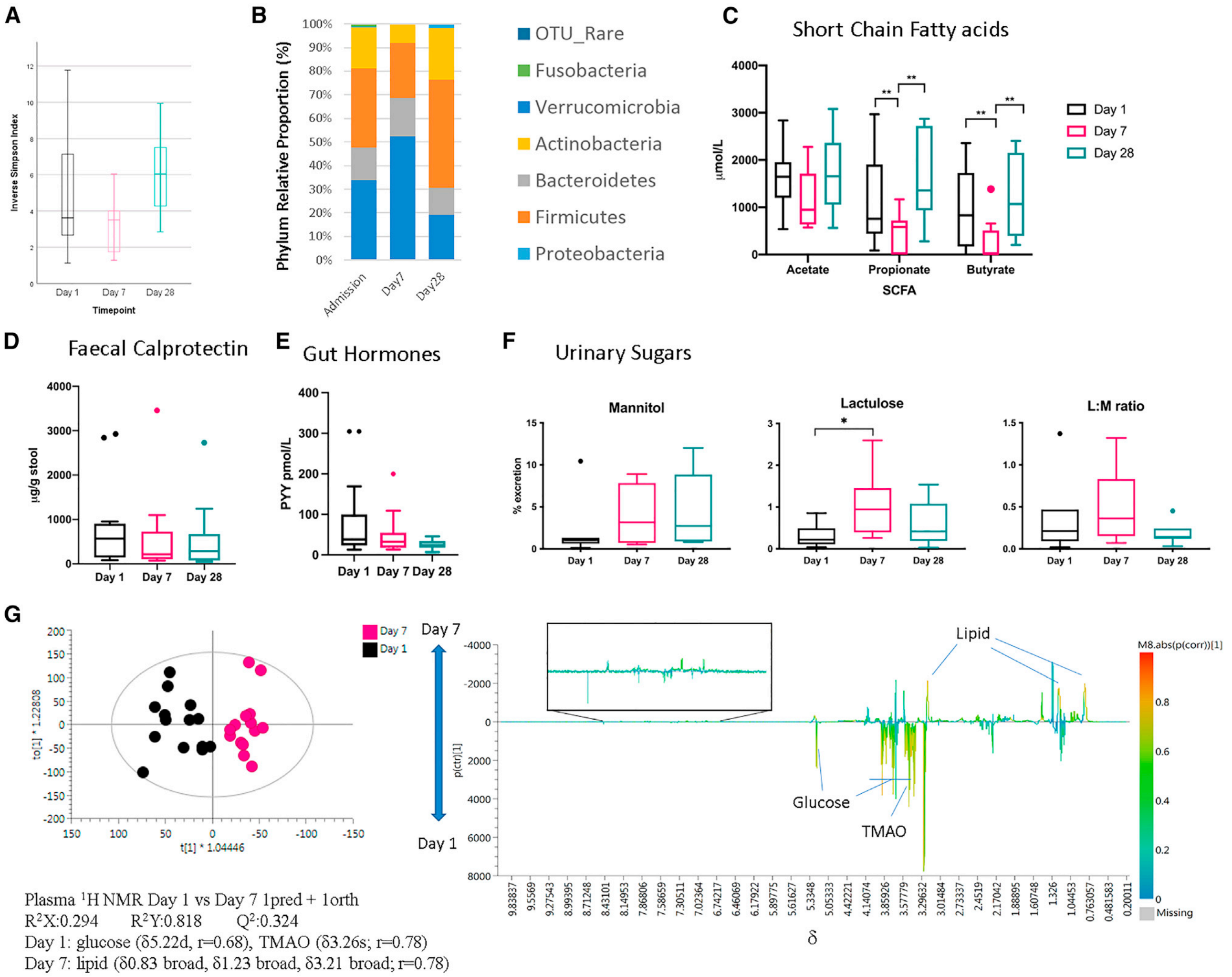

Figure 3. Changes over three time points (day 1, day 7, and day 28) of gut function markers in children receiving ConF (n = 18)

(A and B) Admission 16S rRNA-derived bacterial phyla relative abundances for F75 control arm at each time point demonstrating *Proteobacteria* are higher in days 1 and 7 than in day 28. *Firmicutes* are higher in day 28 than in days 1 and 7, and *Bacteroidetes* are higher in day 28 than in day 7. Day 1, n = 16; day 7, n = 13; day 28, n = 13.

(C) Fecal SCFA concentrations. Days 1/7/28: acetate, n = 13/13/13; propionate, n = 13/13/13; butyrate, n = 13/13/13.

(D) Fecal calprotectin. Days 1/7/28: n = 13/13/13.

(E) Plasma PYY. Days 1/7/28: n = 17/15/14.

(F) L:M ratio. Days 1/7/28: n = 7/7/7.

(G) OPLS-DA (Orthogonal Projection to Latent structures-Discriminant Analysis) scores and loading plots of plasma ^{1}H NMR spectra showing differentiation in metabolite profiles of day 1 to day 7. Days 1/7: n = 14/14.

Comparisons between days were made by t test or ANOVA where relevant.

IQR: 0.24, p = 0.002) before falling again on day 28 (median: 0.41%; lactulose-recovered IQR: 0.53). Our interpretation of these results is that antibiotics initiated on admission decreased colonic microbial diversity, leading to a decrease in SCFA production.[48,49] SFCAs, particularly butyrate, are important in the maintenance of the colonic epithelium. Low concentrations will lead to a decrease in epithelial integrity and an increase in lactulose absorption and excretion on day 7. From days 7 to 28, following the completion of antibiotic treatment, microbial SCFA production increased, resulting in a stimulation of an improved epithelial integrity and a decrease of lactulose absorption and excretion. No significant change was observed in the L:M ratio (Figure 3F). These results show that even in SAM children who survive, there is significant GI dysfunction on admission (high fecal calprotectin and raised L:M ratio), which improved over the 28 days. It is of interest that there was a decrease in SCFA concentrations on day 7, following the commencement of antibiotic therapy.

The plasma metabolomic enquiry demonstrated that there was good separation between days 1 and 7 in the metabolite

profile, driven primarily by glucose elevation at day 1 and a relative increase in lipid by day 7, reflecting either dietary changes or the effect of sepsis and subsequent recovery (Figure 3G). Additionally, trimethylamine (TMA)-N-oxide (TMAO) was high on admission. TMAO is produced in the liver from TMA, a gut metabolite associated with *Proteobacteria*, which is also high on days 1 and 7.[50] TMAO is of interest, as it has been associated with a negative effect on muscle and linear growth.[51]

Impact of CpFs on gut health in comparison to ConFs and InFs

In the measures of intestinal injury and integrity, there was no significant difference in fecal calprotectin (Figure 4A) among the three feeds. Although the dual-sugar test demonstrated a significant effect of time for urinary mannitol excretion (%M, p = 0.0001) (Figure 4B), there was no effect of intervention (p = 0.12) or a significant intervention × time interaction (p = 0.32). There was a significant intervention effect for urinary lactulose excretion (%L, p = 0.024) but no effect of time (p = 0.27) or a significant intervention × time interaction (p = 0.44). There was a significant time effect (L:M, p = 0.006) on the urinary L:M ratio, but there was no effect of intervention (p = 0.18), and there was a trend toward a significant intervention × time interaction (p = 0.063) (Figure 4B). Likewise, there were no significant differences among intervention arms for the gut hormones GLP-1 and PYY (Figure 4C). SCFA (Figure 4D) analysis found few differences among intervention arms for acetate. Butyrate showed a significant decrease at day 7, followed by recovery at day 28 to the level observed at day 1 in the ConF group, while a similar pattern was found for propionate in inulin-treated patients. In contrast, in cowpea-treated patients, this decline was truncated, with no significant changes over time (Figure 4D). We used a commercial source of inulin, a fructan, as a standard non-digestible fermentable carbohydrate to enrich the feeds in one arm (InF). However, we did not observe the same effect as CpFs on SCFAs or amelioration of the reduction in microbial diversity at day 7 with InF, suggesting that digestion/fermentation of this substrate may have been limited (Figure 4D; Table S3). Similar observations have been made with manufactured resistant starches (e.g., hylon, a high-amylose starch derived from maize,[52] results in significant excretion in stool). This may also explain hylon's limited effect when used in malnourished Malawian children.[53]

Analysis of the microbiome demonstrated a significant decrease in microbial richness as calculated by ACE (abundance-based coverage estimator) in the ConF group (day 1 ACE = 55.5 ± SD 20.9; day 7 ACE = 42.2 ± SD 12.9; post-hoc Tukey p = 0.027). A similar but insignificant drop in ACE occurred in the InF group (day 1 ACE = 57.2 ± SD 32.3; day 7 ACE = 35.4 ± SD 12.7; post-hoc Tukey p = 0.108) (Figure 4E; Tables S3 and S4). In the CpF group, the drop in richness from day 1 (ACE = 56.0 ± SD 31.0) to day 7 (ACE = 47.9 ± SD 20.6) was less extreme and statistically insignificant (p = 0.691). No significant differences were observed in *Bifidobacterium* genus relative abundance either over time or among intervention arms (Figure 4F), although by day 28, the CpF group had the highest relative abundance (10.9% ± SD 8.7%) of the three intervention arms (ConF = 8.9% ± SD 10.3%; InF = 5.7% ± SD 5.8%; Kruskal Wallis H-test BH FDR (Benjamini-Hochberg False Discovery Rate method) p = 1.000).

Taken together, these results suggest that the CpF has a positive effect on the fecal microbiota, particularly between days 1 and 7. This is of particular interest, given that antibiotics are started over this period of time. Antibiotic usage is reported in Table S5; however, the only difference observed was that no children in the ConF group received four separate antibiotics, compared to 25% (n = 5/20) of children in the InF arm and 20% (n = 4/20) of children in the CpF arm (p = 0.023) received 4 antibiotics. It also appears that the microbiota functionality may be protected by the CpF with no fall in butyrate.

DISCUSSION

This study adds to the number of reports of undernourished children that have demonstrated a relatively immature fecal microbiota with low microbial diversity,[54,55] leading to production of lower concentrations of SCFAs, which are critically important to epithelial function and integrity. We suspect that the use of antibiotics results in a further lowering of microbial diversity, resulting in lower SCFA concentrations at a critical period of nutritional rehabilitation: in the first week, when mortality rate is highest. Lower levels of stool butyrate and propionate have been observed in children who died from SAM compared to survivors.[15] This observation is shown in the present study, where the children who died had significantly lower stool concentrations of butyrate and propionate than those that survived. Even at day 28, the microbiota diversity was as low as admission, and SCFA concentrations had only recovered to admission concentrations.

In children that died, compared to those that survived, there appeared to be increased levels of gut dysfunction including increased fecal proteolytic bacteria on admission and day 7, together with a significantly higher L:M ratio, suggesting a loss of gut integrity. We also observed that the gut hormone PYY was significantly higher in those that died,[56] suggesting appetite dysregulation. Gut integrity has been shown by others to be related to mortality.[57] Although not fully understood, this may be due in part to a low diversity of microbiota in which pathogens can flourish, resulting in increased risk of toxins and enteric bacterial pathogens crossing the perturbed gut barrier. This is likely to result in an increased risk of gram-negative sepsis,[4,10] as supported by the reduction in gut integrity assessed by the L:M ratio.

It is of interest that children who received the ConF had low bacteria diversity on admission, specifically with low levels of saccharolytic bacteria (*Firmicutes* and *Bacteroidetes*) that relate to the low concentration of fecal SCFAs, which relates to a marker of gut dysfunction. We believe these findings early in admission are further compounded by the routine use of antibiotics. A significantly greater proportion of children in the InF (25%, n = 5/20) and CpF (20%, n = 4/20) received four antibiotics during their inpatient admission, compared to the ConF group (0%, n = 0.18; p = 0.023) (Table S5). While durations and dosages could not be examined methodically, the fact that higher fecal SCFA concentrations and lower L:M recovery and ratios were found in children in the CpF group at day 7 is encouraging. These differences support the hypothesis that provision of a

CellPress
OPEN ACCESS

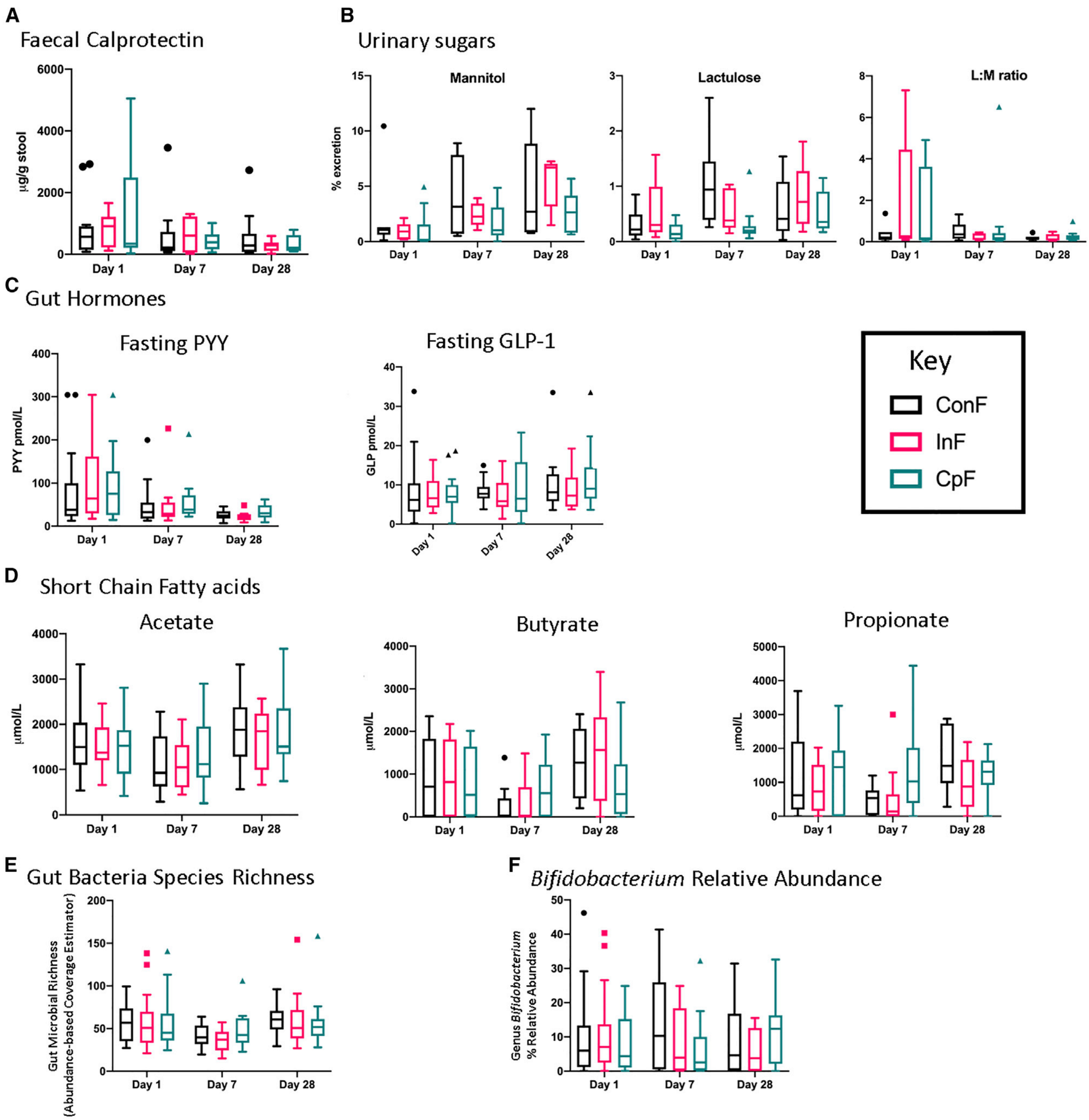

Figure 4. Differences among the three intervention arms over time (days 1, 7, and 28) on markers of gut function

(A) Fecal calprotectin. ConF/InF/CpF: day 1, n = 13/10/15; day 7, n = 13/15/15; day 28, n = 13/10/15.

(B) Mannitol lactulose test. ConF/InF/CpF: day 1, n = 7/5/10; day 7, n = 7/5/10; day 28, n = 7/5/10.

(C) Fasting PYY and GLP-1. ConF/InF/CpF: day 1, n = 17/18/19; day 7, n = 15/12/15; day 28, n = 14/9/14.

(D) Fecal SCFAs. ConF/InF/CpF: day 1, n = 13/15/17; day 7, n = 13/13/15; day 28, n = 13/10/15.

(E) ACE (abundance-based coverage estimator) assessment of fecal bacterial richness. ConF/InF/CpF: day 1, n = 16/19/18; day 7, n = 13/13/15; day 28, n = 13/10/15.

(F) Bifidobacterium relative abundance. Data are presented as median with 95% CL. ConF/InF/CpF: day 1, n = 13/18/17; day 7, n = 13/13/15; day 28, n = 13/10/15.

Comparison between and within intervention arms was carried out using Kruskal-Wallis one-way analysis of variance. Full experimental results are reported in Tables S3 and S4.

fermentable carbohydrate source helps preserve bacterial functionality in the face of antibiotic use.

The current study has evaluated the use of a legume-enriched feed instituted from admission in children hospitalized with severe and complicated malnutrition. We used cowpeas as the supplement to ConF support, as these were locally grown and are a general part of standard diets.[58] In our proof-of-concept study, we demonstrated no adverse effects of the feed, which has resulted in a phase II trial using a food industry standard feed developed by a commercial partner.[59] The children randomized to the CpF gain a similar amount of weight, had increased mid-upper arm circumference, and had no difference in diarrheal episodes compare to ConFs. In fact, our data indicated legume-enriched feeds reduced the impact of antibiotics on further lowering the diversity of the microbiota early in the course of admission/nutritional rehabilitation. Legume-enriched feeds have been used in stable stunted children in the past and are shown to have a positive effect on growth faltering[32] and gut integrity.[30] Further research is warranted.

Limitations of study

The current study is a proof-of-principle trial assessing safety and tolerability of a legume-enriched feeds, compared to ConFs. No formal sample size calculation was possible due to a lack of previous studies reporting on the physiological measures included, such as gut hormones, fecal SCFAs, plasma ^{1}H NMR metabolic profiles, and gut microbial profiles. While weight regain could have been chosen, weight as a clinical outcome has little relation to the physiological markers used to assess the wider effect of feeds. The study was limited in duration to 28 days, so longer-term outcomes of children are not available, which may affect some of the conclusions of this study.

STAR★METHODS

Detailed methods are provided in the online version of this paper and include the following:

- KEY RESOURCES TABLE
- RESOURCE AVAILABILITY
  - Lead contact
  - Materials availability
  - Data and code availability
- EXPERIMENTAL MODEL AND SUBJECT DETAILS
  - Development of cowpea enriched feed
  - Human subjects
- METHOD DETAILS
  - Clinical trial
  - Faecal calprotectin
  - Gut hormones
  - Gas chromatography-mass spectrometry
  - Proton (^{1}H) nuclear magnetic resonance (NMR) spectroscopy
  - 16S rRNA sequencing
- QUANTIFICATION AND STATISTICAL ANALYSIS
  - Statistical analysis
- ADDITIONAL RESOURCES

SUPPLEMENTAL INFORMATION

Supplemental information can be found online at https://doi.org/10.1016/j.xcrm.2021.100280.

ACKNOWLEDGMENTS

We thank all the participants and staff from hospital and research units participating in the MIMBLE study. We would like to thank Siraj Kijogo (Head) and Jennifer Adong (Senior Nurse) of the Mbale Regional Referral Hospital Nutrition Unit. We also thank the medical students Pylin Parkes and Gina Tse for assistance with running the trial. G.F. is an NIHR senior investigator. This study is funded by a fellowship award to N.C. from the Wellcome Trust Institutional Strategic Support Fund (grant code 105603/Z/14/Z). Support for the trial management [KEMRI Wellcome Trust Programme East African Overseas Programme Award (2016) from the Wellcome Trust 203077/Z/16/Z])

AUTHOR CONTRIBUTIONS

Conceptualization, G.F., K.M., and N.C.; formal analysis, N.C., K.W., J.B., X.W., and D.M.; investigation, N.C., A.M., P.O.-O., T.S., R.M., K.W., E.M., D.M., G.F., and E.H.; data curation, N.C. and K.W.; writing – review & editing, K.M., G.F., N.C., K.W., D.M., and P.O.-O.

DECLARATION OF INTERESTS

The authors declare no competing interests.

INCLUSION AND DIVERSITY

We worked to ensure gender balance in the recruitment of human subjects. One or more of the authors of this paper self-identifies as an underrepresented ethnic minority in science. One or more of the authors of this paper self-identifies as a member of the LGBTQ+ community. One or more of the authors of this paper received support from a program designed to increase minority representation in science. While citing references scientifically relevant for this work, we also actively worked to promote gender balance in our reference list. The author list of this paper includes contributors from the location where the research was conducted who participated in the data collection, design, analysis, and/or interpretation of the work.

Received: August 14, 2020
Revised: February 23, 2021
Accepted: April 22, 2021
Published: May 18, 2021

REFERENCES

1. World Health Organization (2017). Children: reducing mortality. 01/10, 2017 ed. (World Health Organization).
2. Black, R.E., Victora, C.G., Walker, S.P., Bhutta, Z.A., Christian, P., de Onis, M., Ezzati, M., Grantham-McGregor, S., Katz, J., Martorell, R., and Uauy, R.; Maternal and Child Nutrition Study Group (2013). Maternal and child undernutrition and overweight in low-income and middle-income countries. Lancet *382*, 427–451.
3. Bhutta, Z.A., Berkley, J.A., Bandsma, R.H.J., Kerac, M., Trehan, I., and Briend, A. (2017). Severe childhood malnutrition. Nat. Rev. Dis. Primers *3*, 17067.
4. Maitland, K., Berkley, J.A., Shebbe, M., Peshu, N., English, M., and Newton, C.R. (2006). Children with severe malnutrition: can those at highest risk of death be identified with the WHO protocol? PLoS Med. *3*, e500.
5. Gachau, S., Irimu, G., Ayieko, P., Akech, S., Agweyu, A., and English, M.; Clinical Information Network Author Group (2018). Prevalence, outcome and quality of care among children hospitalized with severe acute

malnutrition in Kenyan hospitals: A multi-site observational study. PLoS ONE *13*, e0197607.

6. Berkley, J.A., Ngari, M., Thitiri, J., Mwalekwa, L., Timbwa, M., Hamid, F., Ali, R., Shangala, J., Mturi, N., Jones, K.D., et al. (2016). Daily co-trimoxazole prophylaxis to prevent mortality in children with complicated severe acute malnutrition: a multicentre, double-blind, randomised placebo-controlled trial. Lancet Glob. Health *4*, e464–e473.
7. Kerac, M., Bunn, J., Chagaluka, G., Bahwere, P., Tomkins, A., Collins, S., and Seal, A. (2014). Follow-up of post-discharge growth and mortality after treatment for severe acute malnutrition (FuSAM study): a prospective cohort study. PLoS ONE *9*, e96030.
8. World Health Organization. (2013). Updates on the management of severe acute malnutrition in infants and children (WHO).
9. Ngari, M.M., Mwalekwa, L., Timbwa, M., Hamid, F., Ali, R., Iversen, P.O., Fegan, G.W., and Berkley, J.A. (2018). Changes in susceptibility to life-threatening infections after treatment for complicated severe malnutrition in Kenya. Am. J. Clin. Nutr. *107*, 626–634.
10. Talbert, A., Thuo, N., Karisa, J., Chesaro, C., Ohuma, E., Ignas, J., Berkley, J.A., Toromo, C., Atkinson, S., and Maitland, K. (2012). Diarrhoea complicating severe acute malnutrition in Kenyan children: a prospective descriptive study of risk factors and outcome. PLoS ONE *7*, e38321.
11. Bejon, P., Mwangi, I., Ngetsa, C., Mwarumba, S., Berkley, J.A., Lowe, B.S., Maitland, K., Marsh, K., English, M., and Scott, J.A. (2005). Invasive Gram-negative bacilli are frequently resistant to standard antibiotics for children admitted to hospital in Kilifi, Kenya. J. Antimicrob. Chemother. *56*, 232–235.
12. Versloot, C.J., Attia, S., Bourdon, C., Richardson, S.E., Potani, I., Bandsma, R.H.J., and Voskuijl, W. (2018). Intestinal pathogen clearance in children with severe acute malnutrition is unrelated to inpatient morbidity. Clin. Nutr. ESPEN *24*, 109–113.
13. Subramanian, S., Huq, S., Yatsunenko, T., Haque, R., Mahfuz, M., Alam, M.A., Benezra, A., DeStefano, J., Meier, M.F., Muegge, B.D., et al. (2014). Persistent gut microbiota immaturity in malnourished Bangladeshi children. Nature *510*, 417–421.
14. Jones, K.D., Hünten-Kirsch, B., Laving, A.M., Munyi, C.W., Ngari, M., Mikusa, J., Mulongo, M.M., Odera, D., Nassir, H.S., Timbwa, M., et al. (2014). Mesalazine in the initial management of severely acutely malnourished children with environmental enteric dysfunction: a pilot randomized controlled trial. BMC Med. *12*, 133.
15. Attia, S., Versloot, C.J., Voskuijl, W., van Vliet, S.J., Di Giovanni, V., Zhang, L., Richardson, S., Bourdon, C., Netea, M.G., Berkley, J.A., et al. (2016). Mortality in children with complicated severe acute malnutrition is related to intestinal and systemic inflammation: an observational cohort study. Am. J. Clin. Nutr. *104*, 1441–1449.
16. Muenchhoff, M., Healy, M., Singh, R., Roider, J., Groll, A., Kindra, C., Sibaya, T., Moonsamy, A., McGregor, C., Phan, M.Q., et al. (2018). Malnutrition in HIV-Infected Children Is an Indicator of Severe Disease with an Impaired Response to Antiretroviral Therapy. AIDS Res. Hum. Retroviruses *34*, 46–55.
17. Ramakrishna, B.S. (2013). Role of the gut microbiota in human nutrition and metabolism. J. Gastroenterol. Hepatol. *28*, 9–17.
18. Wu, Y., Wan, J., Choe, U., Pham, Q., Schoene, N.W., He, Q., Li, B., Yu, L., and Wang, T.T.Y. (2019). Interactions Between Food and Gut Microbiota: Impact on Human Health. Annu. Rev. Food Sci. Technol. *10*, 389–408.
19. Martin, F.P., Wang, Y., Sprenger, N., Yap, I.K., Lundstedt, T., Lek, P., Rezzi, S., Ramadan, Z., van Bladeren, P., Fay, L.B., et al. (2008). Probiotic modulation of symbiotic gut microbial-host metabolic interactions in a humanized microbiome mouse model. Mol. Syst. Biol. *4*, 157.
20. Pekmez, C.T., Dragsted, L.O., and Brahe, L.K. (2019). Gut microbiota alterations and dietary modulation in childhood malnutrition - The role of short chain fatty acids. Clin. Nutr. *38*, 615–630.
21. Maccaferri, S., Klinder, A., Cacciatore, S., Chitarrari, R., Honda, H., Luchinat, C., Bertini, I., Carnevali, P., Gibson, G.R., Brigidi, P., and Costabile, A. (2012). In vitro fermentation of potential prebiotic flours from natural sources: impact on the human colonic microbiota and metabolome. Mol. Nutr. Food Res. *56*, 1342–1352.
22. Martín-Peláez, S., Gibson, G.R., Martín-Orúe, S.M., Klinder, A., Rastall, R.A., La Ragione, R.M., Woodward, M.J., and Costabile, A. (2008). In vitro fermentation of carbohydrates by porcine faecal inocula and their influence on Salmonella Typhimurium growth in batch culture systems. FEMS Microbiol. Ecol. *66*, 608–619.
23. Roberfroid, M., Gibson, G.R., Hoyles, L., McCartney, A.L., Rastall, R., Rowland, I., Wolvers, D., Watzl, B., Szajewska, H., Stahl, B., et al. (2010). Prebiotic effects: metabolic and health benefits. Br. J. Nutr. *104*, S1–S63.
24. Gehrig, J.L., Venkatesh, S., Chang, H.W., Hibberd, M.C., Kung, V.L., Cheng, J., Chen, R.Y., Subramanian, S., Cowardin, C.A., Meier, M.F., et al. (2019). Effects of microbiota-directed foods in gnotobiotic animals and undernourished children. Science *365*, eaau4732.
25. Anyango, J.O., de Kock, H.L., and Taylor, J.R.N. (2011). Impact of cowpea addition on the Protein Digestibility Corrected Amino Acid Score and other protein quality parameters of traditional African foods made from non-tannin and tannin sorghum. Food Chem. *124*, 775–780.
26. Eshwarage, I., Herath, T., and Gunathilake, T. (2017). Dietary fibre, resistant starch and in-vitro starch digestibility of selected commonly consumed legumes (mung bean, cowpea, soybean and horse gram) in Sri Lanka. Res. J. Chem. Sci. *7*, 27–33.
27. Mallillin, A.C., Trinidad, T.P., Raterta, R., Dagbay, K., and Loyola, A.S. (2008). Dietary fibre and fermentability characteristics of root crops and legumes. Br. J. Nutr. *100*, 485–488.
28. Guillon, F., and Champ, M.M. (2002). Carbohydrate fractions of legumes: uses in human nutrition and potential for health. Br. J. Nutr. *88*, S293–S306.
29. Noah, L., Guillon, F., Bouchet, B., Buléon, A., Molis, C., Gratas, M., and Champ, M. (1998). Digestion of carbohydrate from white beans (Phaseolus vulgaris L.) in healthy humans. J. Nutr. *128*, 977–985.
30. Agapova, S.E., Stephenson, K.B., Divala, O., Kaimila, Y., Maleta, K.M., Thakwalakwa, C., Ordiz, M.I., Trehan, I., and Manary, M.J. (2018). Additional Common Bean in the Diet of Malawian Children Does Not Affect Linear Growth, but Reduces Intestinal Permeability. J. Nutr. *148*, 267–274.
31. Ordiz, M.I., Janssen, S., Humphrey, G., Ackermann, G., Stephenson, K., Agapova, S., Divala, O., Kaimila, Y., Maleta, K., Zhong, C., et al. (2020). The effect of legume supplementation on the gut microbiota in rural Malawian infants aged 6 to 12 months. Am. J. Clin. Nutr. *111*, 884–892.
32. Stephenson, K.B., Agapova, S.E., Divala, O., Kaimila, Y., Maleta, K.M., Thakwalakwa, C., Ordiz, M.I., Trehan, I., and Manary, M.J. (2017). Complementary feeding with cowpea reduces growth faltering in rural Malawian infants: a blind, randomized controlled clinical trial. Am. J. Clin. Nutr. *106*, 1500–1507.
33. Williams, P.C.M., and Berkley, J.A. (2018). Guidelines for the treatment of severe acute malnutrition: a systematic review of the evidence for antimicrobial therapy. Paediatr. Int. Child Health *38*, S32–S49.
34. Yoon, M.Y., and Yoon, S.S. (2018). Disruption of the Gut Ecosystem by Antibiotics. Yonsei Med. J. *59*, 4–12.
35. Francino, M.P. (2016). Antibiotics and the Human Gut Microbiome: Dysbioses and Accumulation of Resistances. Front. Microbiol. *6*, 1543.
36. World Health Organisation (2003). Guidelines for the inpatient treatment of severely malnourished children (World Health Organization), p. 49.
37. Pham, T.P., Tidjani Alou, M., Bachar, D., Levasseur, A., Brah, S., Alhousseini, D., Sokhna, C., Diallo, A., Wieringa, F., Million, M., and Raoult, D. (2019). Gut Microbiota Alteration is Characterized by a Proteobacteria and Fusobacteria Bloom in Kwashiorkor and a Bacteroidetes Paucity in Marasmus. Sci. Rep. *9*, 9084.

38. Wong, J.M., de Souza, R., Kendall, C.W., Emam, A., and Jenkins, D.J. (2006). Colonic health: fermentation and short chain fatty acids. J. Clin. Gastroenterol. *40*, 235–243.

39. den Besten, G., van Eunen, K., Groen, A.K., Venema, K., Reijngoud, D.J., and Bakker, B.M. (2013). The role of short-chain fatty acids in the interplay between diet, gut microbiota, and host energy metabolism. J. Lipid Res. *54*, 2325–2340.

40. Nofrarías, M., Martínez-Puig, D., Pujols, J., Majó, N., and Pérez, J.F. (2007). Long-term intake of resistant starch improves colonic mucosal integrity and reduces gut apoptosis and blood immune cells. Nutrition *23*, 861–870.

41. Knock, G., Psaroudakis, D., Abbot, S., and Aaronson, P.I. (2002). Propionate-induced relaxation in rat mesenteric arteries: a role for endothelium-derived hyperpolarising factor. J Physiol *538*, 879–890. https://doi.org/10.1113/jphysiol.2001.013105.

42. Denno, D.M., VanBuskirk, K., Nelson, Z.C., Musser, C.A., Hay Burgess, D.C., and Tarr, P.I. (2014). Use of the lactulose to mannitol ratio to evaluate childhood environmental enteric dysfunction: a systematic review. Clin. Infect. Dis. *59*, S213–S219.

43. Moran, G.W., Leslie, F.C., and McLaughlin, J.T. (2013). Crohn's disease affecting the small bowel is associated with reduced appetite and elevated levels of circulating gut peptides. Clin. Nutr. *32*, 404–411.

44. Smith, M.I., Yatsunenko, T., Manary, M.J., Trehan, I., Mkakosya, R., Cheng, J., Kau, A.L., Rich, S.S., Concannon, P., Mychaleckyj, J.C., et al. (2013). Gut microbiomes of Malawian twin pairs discordant for kwashiorkor. Science *339*, 548–554.

45. Morrison, D.J., and Preston, T. (2016). Formation of short chain fatty acids by the gut microbiota and their impact on human metabolism. Gut Microbes *7*, 189–200.

46. De Filippo, C., Cavalieri, D., Di Paola, M., Ramazzotti, M., Poullet, J.B., Massart, S., Collini, S., Pieraccini, G., and Lionetti, P. (2010). Impact of diet in shaping gut microbiota revealed by a comparative study in children from Europe and rural Africa. Proc. Natl. Acad. Sci. USA *107*, 14691–14696.

47. Kane, A.V., Dinh, D.M., and Ward, H.D. (2015). Childhood malnutrition and the intestinal microbiome. Pediatr. Res. *77*, 256–262.

48. Reijnders, D., Goossens, G.H., Hermes, G.D., Neis, E.P., van der Beek, C.M., Most, J., Holst, J.J., Lenaerts, K., Kootte, R.S., Nieuwdorp, M., et al. (2016). Effects of Gut Microbiota Manipulation by Antibiotics on Host Metabolism in Obese Humans: A Randomized Double-Blind Placebo-Controlled Trial. Cell Metab. *24*, 341.

49. Mellon, A.F., Deshpande, S.A., Mathers, J.C., and Bartlett, K. (2000). Effect of oral antibiotics on intestinal production of propionic acid. Arch. Dis. Child. *82*, 169–172.

50. Rath, S., Heidrich, B., Pieper, D.H., and Vital, M. (2017). Uncovering the trimethylamine-producing bacteria of the human gut microbiota. Microbiome *5*, 54.

51. Semba, R.D., Zhang, P., Gonzalez-Freire, M., Moaddel, R., Trehan, I., Maleta, K.M., Ordiz, M.I., Ferrucci, L., and Manary, M.J. (2016). The association of serum choline with linear growth failure in young children from rural Malawi. Am. J. Clin. Nutr. *104*, 191–197.

52. van Munster, I.P., Tangerman, A., and Nagengast, F.M. (1994). Effect of resistant starch on colonic fermentation, bile acid metabolism, and mucosal proliferation. Dig. Dis. Sci. *39*, 834–842.

53. Ordiz, M.I., May, T.D., Mihindukulasuriya, K., Martin, J., Crowley, J., Tarr, P.I., Ryan, K., Mortimer, E., Gopalsamy, G., Maleta, K., et al. (2015). The effect of dietary resistant starch type 2 on the microbiota and markers of gut inflammation in rural Malawi children. Microbiome *3*, 37.

54. Gough, E.K., Moodie, E.E., Prendergast, A.J., Johnson, S.M., Humphrey, J.H., Stoltzfus, R.J., Walker, A.S., Trehan, I., Gibb, D.M., Goto, R., et al. (2014). The impact of antibiotics on growth in children in low and middle income countries: systematic review and meta-analysis of randomised controlled trials. BMJ *348*, g2267.

55. Monira, S., Nakamura, S., Gotoh, K., Izutsu, K., Watanabe, H., Alam, N.H., Endtz, H.P., Cravioto, A., Ali, S.I., Nakaya, T., et al. (2011). Gut microbiota of healthy and malnourished children in bangladesh. Front. Microbiol. *2*, 228.

56. Bartz, S., Mody, A., Hornik, C., Bain, J., Muehlbauer, M., Kiyimba, T., Kiboneka, E., Stevens, R., Bartlett, J., St Peter, J.V., et al. (2014). Severe acute malnutrition in childhood: hormonal and metabolic status at presentation, response to treatment, and predictors of mortality. J. Clin. Endocrinol. Metab. *99*, 2128–2137.

57. Otani, S., and Coopersmith, C.M. (2019). Gut integrity in critical illness. J. Intensive Care *7*, 17.

58. Oyeleke, O.A., Morton, I.D., and Bender, A.E. (1985). The use of cowpeas (Vigna unguiculata) in improving a popular Nigerian weaning food. Br. J. Nutr. *54*, 343–347.

59. Walsh, K., Caldar, N., Olupot-Olupot, P., Ssenyondo, T., Okiri, W., Okalebo, C.B., et al. (2018). Modifying Intestinal Integrity and MicroBiome in Severe Malnutrition with Legume-Based Feeds (MIMBLE 2.0): protocol for a phase II refined feed and intervention trial (Wellcome Open).

60. Kunz, C., Rudloff, S., Baier, W., Klein, N., and Strobel, S. (2000). Oligosaccharides in human milk: structural, functional, and metabolic aspects. Annu. Rev. Nutr. *20*, 699–722.

61. Olano-Martin, E., Mountzouris, K.C., Gibson, G.R., and Rastall, R.A. (2000). In vitro fermentability of dextran, oligodextran and maltodextrin by human gut bacteria. Br. J. Nutr. *83*, 247–255.

62. Generoso, M., De Rosa, M., De Rosa, R., De Magistris, L., Secondulfo, M., Fiandra, R., Carratù, R., and Cartenì, M. (2003). Cellobiose and lactulose coupled with mannitol and determined using ion-exchange chromatography with pulsed amperometric detection, are reliable probes for investigation of intestinal permeability. J. Chromatogr. B Analyt. Technol. Biomed. Life Sci. *783*, 349–357.

63. Adrian, T.E., Ferri, G.L., Bacarese-Hamilton, A.J., Fuessl, H.S., Polak, J.M., and Bloom, S.R. (1985). Human distribution and release of a putative new gut hormone, peptide YY. Gastroenterology *89*, 1070–1077.

64. Kreymann, B., Williams, G., Ghatei, M.A., and Bloom, S.R. (1987). Glucagon-like peptide-1 7-36: a physiological incretin in man. Lancet *2*, 1300–1304.

65. García-Villalba, R., Giménez-Bastida, J.A., García-Conesa, M.T., Tomás-Barberán, F.A., Carlos Espín, J., and Larrosa, M. (2012). Alternative method for gas chromatography-mass spectrometry analysis of short-chain fatty acids in faecal samples. J. Sep. Sci. *35*, 1906–1913.

66. Dona, A.C., Jiménez, B., Schäfer, H., Humpfer, E., Spraul, M., Lewis, M.R., Pearce, J.T., Holmes, E., Lindon, J.C., and Nicholson, J.K. (2014). Precision high-throughput proton NMR spectroscopy of human urine, serum, and plasma for large-scale metabolic phenotyping. Anal. Chem. *86*, 9887–9894.

67. Mullish, B.H., Pechlivanis, A., Barker, G.F., Thursz, M.R., Marchesi, J.R., and McDonald, J.A.K. (2018). Functional microbiomics: Evaluation of gut microbiota-bile acid metabolism interactions in health and disease. Methods *149*, 49–58.

68. Cloarec, O., Dumas, M.E., Craig, A., Barton, R.H., Trygg, J., Hudson, J., Blancher, C., Gauguier, D., Lindon, J.C., Holmes, E., and Nicholson, J. (2005). Statistical total correlation spectroscopy: an exploratory approach for latent biomarker identification from metabolic 1H NMR data sets. Anal. Chem. *77*, 1282–1289.

69. Posma, J.M., Garcia-Perez, I., De Iorio, M., Lindon, J.C., Elliott, P., Holmes, E., Ebbels, T.M., and Nicholson, J.K. (2012). Subset optimization by reference matching (STORM): an optimized statistical approach for recovery of metabolic biomarker structural information from 1H NMR spectra of biofluids. Anal. Chem. *84*, 10694–10701.

70. Wishart, D.S., Feunang, Y.D., Marcu, A., Guo, A.C., Liang, K., Vázquez-Fresno, R., Sajed, T., Johnson, D., Li, C., Karu, N., et al. (2018). HMDB 4.0: the human metabolome database for 2018. Nucleic Acids Res. *46*, D608–D617.

CellPress
OPEN ACCESS

71. Quast, C., Pruesse, E., Yilmaz, P., Gerken, J., Schweer, T., Yarza, P., Peplies, J., and Glöckner, F.O. (2013). The SILVA ribosomal RNA gene database project: improved data processing and web-based tools. Nucleic Acids Res. *41*, D590–D596.

72. DeSantis, T.Z., Hugenholtz, P., Larsen, N., Rojas, M., Brodie, E.L., Keller, K., Huber, T., Dalevi, D., Hu, P., and Andersen, G.L. (2006). Greengenes, a chimera-checked 16S rRNA gene database and workbench compatible with ARB. Appl. Environ. Microbiol. *72*, 5069–5072.

73. Cole, J.R., Wang, Q., Fish, J.A., Chai, B., McGarrell, D.M., Sun, Y., Brown, C.T., Porras-Alfaro, A., Kuske, C.R., and Tiedje, J.M. (2014). Ribosomal Database Project: data and tools for high throughput rRNA analysis. Nucleic Acids Res. *42*, D633–D642.

STAR★METHODS

KEY RESOURCES TABLE

REAGENT or RESOURCE	SOURCE	IDENTIFIER
Chemicals, peptides, and recombinant proteins		
NaH_2PO_4	Sigma-Aldrich	255793; CAS 7558-79-4
TSP	Sigma-Aldrich	613150; CAS 284664-85-3
D_2O	Sigma-Aldrich	1.13366; CAS 7789-20-0
Critical commercial assays		
QIAmp Powerfecal DNA	QIAGEN	Model 11993
QuBit DNA quantification assay	ThermoFisher Scientific	Q32851
MiSeq Reagent Kit v3	Illumina	MS-102-3003
Deposited data		
Mendeley Data, repository of raw data	This study	https://doi.org/10.17632/2w9kjrb682
Recombinant DNA		
Forward primers 28F-YM, 28F-Borrelia, 28FChloroflex, 28F-Bifdo)	Mullish et al., 2018	N/A
Reverse primer 388R	Mullish et al., 2018	N/A
Software and algorithms		
STORM MATLAB script	Posma et al., 2012	https://bitbucket.org/jmp111/storm/src/master/
Topspin v3.1	Bruker, Karlsruhe Gemany	https://www.bruker.com/en/products-and-solutions/mr/nmr-software.html
SIMCA P+	umetrics, Umea Sweden	https://www.sartorius.com/en/products/process-analytical-technology/data-analytics-software/mvda-software/simca
Mothur	Kozich et al., 2013	RRID:SCR_011947
STAMP	Parks et al., 2014	RRID:SCR_018887
Agilent Mass Hunter Software	Agilent	RRID:SCR_015040
Silva	Quast et al., 2013	https://www.arb-silva.de/
RDP	Cole et al., 2014	https://rdp.cme.msu.edu/
Survminer R package		https://cran.r-project.org/web/packages/survminer/index.html
Survival R package		https://github.com/therneau/survival
Other		
F75/F100 feed	Nutriset	https://www.nutriset.fr/en/products
Mixed-bed ion exchange resin	Sigma-Aldrich	D2572
GC-MS column (fused silica capillary column DB-WAXetr)	Agilent	https://www.agilent.com/en/product/gc-columns/wax-gc-columns/db-waxetr-columns#productdetails
Bruker 600Mhz Spectrometer	Bruker, Karlsruhe Germany	https://www.bruker.com/en/products-and-solutions/mr/nmr.html
Orafti Synergy1	BENEO GmbH, Mannheim Germany	https://www.beneo.com/ingredients/human-nutrition/functional-fibres

RESOURCE AVAILABILITY

Lead contact

Further information and requests for resources and reagents should be directed to and will be fulfilled by the lead contact: Professor Kathryn Maitland (k.maitland@imperial.ac.uk)

Materials availability

This study did not generate new unique reagents

Data and code availability

The datasets generated during this study are available at Mendeley Data (https://data.mendeley.com/datasets/2w9kjrb682/2).

EXPERIMENTAL MODEL AND SUBJECT DETAILS

Development of cowpea enriched feed

Our aim was to source a locally grown legume rich in fermentable carbohydrate, milled into a flour that could be used to supplement standard undernutrition recovery feeds F75 and F100 (detailed recipes outlined in supplementary information) to supply 0.5 g of resistant starch/100ml similar to that gained from fermentable oligosaccharide content of breast milk.[60] First, we investigated the impact of cowpea flour and cowpea flour enriched feed on faecal microbiota in-vitro as described in Olano-Martin et al.[61] To do this we undertook batch culture investigation (Figures 1A and 1B). Briefly, 10% faecal slurry was prepared, 10% of which was inoculated into the fermenter alongside ConF, InF and CpF feeds and inulin/cowpea flour separately for 1% final concentration. This was run in triplicate with 3 different faecal donors (who had not had antibiotics for at least 6 months prior to the donation). Samples were fixed in 4% paraformaldehyde, then washed with phosphate-buffered saline (PBS), then stored in 1:1 PBS/ethanol in −20. Total bacteria was determined by fluorescence *in situ* hybridization. We found an increase in microbial number and *Bifidobacteria*, which are thought to have a beneficial impact on health, when milled cowpea and cowpea supplemented feed was added to the batch culture.

Human subjects

Trial design

Modifying Intestinal Integrity and Microbiome in Malnutrition with Legume-Based Feeds [MIMBLE] was a single center (Mbale Regional Referral Hospital) open-label, proof-of-principle randomized comparator trial evaluating safety and feasibility of three feeding strategies. Between 25th January and 26th April 2016 we screened 69 children and enrolled 58 children within 24 hours of hospital admission following informed consent and followed for 28 days (Figure S1). A formal sample size calculation was not conducted but 20 patients per feeding strategy were felt to provide sufficient numbers to assess safety and preliminary data on physiological measurement. The co-primary outcome was weight gain (moderate to good: > 5g/kg/day) and survival to Day 28. Clinical secondary outcomes included time to diarrhea resolution (if > 3 loose stools/day), edema resolution and nutritional rehabilitation. Physiological endpoints included changes in intestinal biomarkers: (i)Intestinal permeability (dual sugar test), intestinal cell injury (faecal calprotectin) (ii) Microbiota: % change in gut microbial flora (iii) Metabolomics: changes in generation of short chain fatty acids and host and microbiota metabolic products and (iv) Gut hormone profile: change in response to feed.

Patient eligibility

Children were eligible for inclusion if they were aged > 6 months < 60months (5years) and had one or more signs of severe acute malnutrition (mid-upper arm circumference (MUAC) < 11.5cm, weight-for-height Z-score (WHZ) < −3 or kwashiorkor (bilateral pedal to generalized edema).[36] Critically sick children with severe malnutrition complicated by impaired consciousness, shock, severe dehydration or infants exclusively breastfed were excluded from the trial. Baseline characteristics are summarized in Table S1. The protocol was approved by the ethics committees of Imperial College London (15IC3006) and Mbale Regional Referral Hospital (UG-IRC-012). The trial was conducted to the standards of ICH Good Clinical Practice.

METHOD DETAILS

Clinical trial

Trial medication: Feed preparation

All nutritional feeds were prepared every 12 hours and stored in a refrigerator (2-8°C) until use. F75/F100 were prepared according to packet instructions with sterile water as described in the following link: https://motherchildnutrition.org/malnutrition-management/info/feeding-formulas-f75-f100.html (accessed May 2020). For InF, a commercial source of mixed chain length inulin (2-60, Orafti Synergy1, BENEO GmbH, Mannheim Germany), was added to F75/F100 packet formulations at 4.8g per liter. CpF was made by adding 35 g/liter cowpea flour to defined amounts of full cream milk powder, sugar, water and vegetable oil to represent F75/F100. The mixture was simmered for 30 minutes. Once cool 20mls/liter mineral mix was added.

Trial procedures and blinding

Fifty-eight children were randomized on a 1:1:1 ratio to WHO nutritional recovery feeds (F75 followed by F100 milk (Nutriset, France) as standard control feeds (ConF n = 18) against standard nutritional feeds enriched with cowpea flour (CpF n = 20) and standard recovery feeds with added standard volume of inulin as positive fermentable carbohydrate control (InF n = 20). Children were fed 3-hourly at a set volume based on their weight, type of malnutrition (non-oedematous versus oedematous) and stage of nutritional rehabilitation as per standard WHO in-hospital SAM management. Feeds were observed by study staff and any balance remaining was recorded for compliance monitoring. All other indicated treatments, including routine antimicrobial therapy, were provided and were the same across all intervention arms. Nurses/doctors were unblinded; all laboratory investigations were assayed blinded.

Baseline anthropometric and clinical characteristics are presented in Table S1. Median age was 18 months [interquartile range 13-23] and anthropometric measurements were balanced between intervention arms, with 37 (64%) children overall presenting with edematous malnutrition. A significantly higher prevalence of diarrhea on trial admission in those randomized to cowpea milk feeds (9/19 (47%) versus 4/38 (11%) in other intervention arms) precluded the use of its resolution as secondary endpoint.

Clinical monitoring

Weight, MUAC and edema score were recorded daily during admission and at day 28. Clinical observations were recorded at set time-points. On admission to day 2, these comprised of 8 hourly pulse rate, respiratory rate, oxygen saturations, axillary temperature, blood glucose (except day 2 when only two measurements were taken) and a standardized questionnaire detailing clinical condition. From day 3 onward observations became 12 hourly pulse rate, respiratory rate, oxygen saturations, axillary temperature and questions on clinical condition. Antibiotic treatments that were received are summarized in Table S5.

Sample collection and lactulose: Mannitol (dual sugar) test

On admission/day 1, 7 and 28 plasma (for gut hormones, metabolomics (with matched urine samples)) and stools (for faecal calprotectin, SCFAs and 16S rRNA analysis) were collected, aliquoted and stored at −80°C. On days 1,7 and 28 children received 2ml/kg of L:M solution (containing 250mg/ml lactulose and 50mg/ml mannitol (stored 2°C −8°C)). Urine was collected in a urine collection bag for one hour prior to L:M solution administration (pre-LM urine sample). L:M solution administration was immediately followed by a feed. Participants then fasted for 5 hours (blood glucose monitored during this period) and all urine passed during this time was collected in a urine collection bag at each void (post-LM urine samples). Urine was immediately stored at −80°C. Simultaneously the gut hormones (PYY and GLP-1) pre-feed plasma sample was obtained approximately 15 minutes prior to the end of the 5 hour fast and the post-feed sample approximately 1.5 hours after feed completion. Post-LM urine samples were homogenized at a later date which involved one freeze-thaw cycle, vortexing for 30 seconds to ensure mixing, centrifuging at 400 g for 5 minutes and then aliquoting into two 2ml samples to be stored at −80°C. Plasma was extracted from blood samples and stored at −80°C.

All samples were transferred to Imperial College for further analysis.

Urine aliquots were transferred to SUERC, University of Glasgow for LM analysis. Using a modified anion exchange chromatography coupled with pulsed amperometry detection (ThermorFisher Scientific Dionex ICS3000 with CarboPac PA20 Analytical (3 × 150 mm), Eluent Gradient: 10 mM OH from −7 to 1 min, 10–30 mM KOH from 1–9 min, 30–35 mM KOH from 9–16 min, Flow Rate: 0.5 mL/min, pulsed amperometric, disposable Au on PTFE electrode (carbohydrate 4-Potential Waveform));[62] lactulose and mannitol were quantified using a combination of internal and external standard calibration. Briefly, internal standard (cellobiose, 20 mM, 50 uL) was added to 1 mL thawed urine. An aliquot of this stock urine (60 μL) was diluted with ultrapure water to 1500 μL total volume and 50 mg of mixed-bed ion exchange resin (Sigma-Aldrich, Gillingham, UK) added to remove interfering ions. Finally, samples were spun (400 x g) and the supernatant decanted to a clean vial for analysis. Concomitantly, the same internal standard was added to a set of external standards containing lactulose and mannitol (six-level calibration, range 0-5mM). Urine sample lactulose:cellobiose and mannitol:cellobiose area ratio was used against the external standard curve lactulose:cellobiose and mannitol:cellobiose area ratio (blank corrected mannitol R^2 = 0.998 and lactulose R^2 = 0.992) to calculate %L, %M and L:M using individual sugar dose and appropriate dilution corrections.

Faecal calprotectin

Faecal Calprotectin was measured using a standard by standard enzyme-linked immunosorbent assay (Charing Cross Hospital, Imperial College Healthcare NHS Trust, London). Only participants completing the study with three time points or day 0/1 and day 28 were analyzed.

Gut hormones

Plasma PYY[63] and GLP-1[64] were measured using a sensitive and specific in-house radio immunoassay at Imperial College, London

Gas chromatography-mass spectrometry

Stool short chain fatty acid SCFA (propionate, butyrate and acetic acid) concentrations were analyzed using a gas chromatography system Agilent 7890A GC (Agilent Technologies, Palo Alto, CA, USA) fitted with a high polarity, polyethylene glycol, fused silica capillary column DB-WAXetr ((30 m, 0.25 mm id, 0.25 μm m film thickness) Agilent Technologies 122-7332LTM). The GC system was coupled to a mass spectrometer system Agilent 5977A, single quadrupole detector with an EI source at 70 eV. Scanning the 30–250 m/z range. The ion source temperature was 230°C, single quadrupole temperature was 150°C and transfer line temperature was 280°C. A solvent delay of 3.5 min was used. The target ions of the single ion monitoring (SIM) employed for this method were: acetic acid-60 m/z, propionic acid-74 m/z and butyric acid- 73 m/z. Identification of the SCFAs was based on the retention time of standard compounds. Stool samples were processed according to published methods.[65]

Proton (^{1}H) nuclear magnetic resonance (NMR) spectroscopy

The methods for sample preparation for NMR spectroscopy have been previously described.[66] Plasma buffer (0.075M NaH2PO4) was prepared as follows and stored at 4°C: 5.32 g of NaH_2PO_4 was dissolved in 380ml of water. 0.4g of reference compound (TSP) was added to this solution and dissolved. 5ml of 4% NaN_3 solution was added and mixed, then 100ml of D_2O added. pH was adjusted to 7.4 using 1M HCl solution. The solution was transferred to a 500ml flask and volume adjusted to 500ml with water.

Samples were thawed and vortex mixed, then centrifuged at 12000 g for 5 minutes at 4°C. 350μl of sample and 350μl of plasma buffer were added to a microcentrifuge tube that was then vortex mixed and centrifuged for 12000 g at 4°C for 5 minutes. 600μl of supernatant was transferred into a SampleJet 5mm NMR tube (Bruker, Karlsruhe Germany), then sealed with POM balls. NMR spectroscopy was conducted using a Bruker 600Mhz Avance Spectrometer (Bruker, Karlsruhe Germany). A ^{1}H 1-dimensional (1D) profile was acquired for each sample using a standard 1D pulse sequence employing the first increment of a Nuclear Overhauser Effect pulse sequence to achieve pre-saturation of the water resonance, followed by a Carr-Purcell-Meiboom-Gill (CPMG) sequence experiment. A 2-dimensional (2D) J-res experiment was also acquired to exploit the structural properties and help with biomarker identification.

16S rRNA sequencing

QIAamp PowerFecal DNA Kits (QIAGEN, Hilden Germany) were used for faecal DNA extraction. QIAamp PowerFecal DNA Kits (QIAGEN, Hilden Germany) were used to extract faecal DNA as described in the protocol. Briefly, inside a biosafety cabinet, 250mg of defrosted faecal sample was transferred into a safe-lock microcentrifuge tube. To this glass beads were added and then 750μl of bead solution and 60μl of solution C1. Tubes were then heated at 65°C for 10minutes, and placed into the Bullet Blender Storm instrument (Next Advance Inc., New York USA), and bead beaten for 3 minutes at speed 8. Tubes were removed and centrifuged for 1 minutes at 13,000 g, after which supernatant was transferred to a clean microcentrifuge tube. 250μl of solution C2 was added to the supernatant, then vortex mixed, and incubated at 4°C for 5 minutes. The tubes were then re-centrifuged at 13,000 g for 1 minute, and up to 750μl of supernatant transferred to a new tube, while avoiding the pellet. 1200μl of solution C4 was added to the supernatant and then vortex mixed. The mixture was loaded onto a spin filter and centrifuged at 13,000 g for 1 minute in 3 × 650μl batches discarding the flow through until all had been filtered. 500μl of solution C5 was then loaded onto the filter, and centrifuged at 13,000 g for 1 minute. The extracted DNA was then eluted from the filter by adding 100μl of solution C6, and centrifuging for 1 minute at 13,000 g. Aliquots of 25μl volume of the eluted DNA was transferred to new tubes and stored at −80°C.

Prior to sequencing, the concentration of DNA was determined using Qubit dsDNA BR assay kits (Thermo Fisher Scientific, Massachusetts USA) as per the protocol, and where necessary DNA was diluted to below 5μg/ml. Sample libraries were prepared according to Illumina's protocol. Illumina 2017. 16S Metagenomic Sequencing Library Preparation Available: https://support.illumina.com/downloads/16s_metagenomic_sequencing_library_preparation.html [Accessed 10/1/2017 2017]. The forward primers (28F-YM, 28F-Borrellia, 28FChloroflex, 28F-Bifdo) were in a mix with a ratio of 4:1:1:1, and the reverse primer used was 388R.[67]

QUANTIFICATION AND STATISTICAL ANALYSIS

Statistical analysis

Statistical analyses were conducted using GraphPad Prism software (version 8.1.2) or R (version 3.6.2). Data are presented as mean ± standard deviation (s.d.) or median ± interquartile range (IQR), as stated. Comparisons between two groups were performed via a t test or Mann-Whitney test, as appropriate. Faecal calprotectin, gut hormone, urine sugar and SCFA data were analyzed using mixed-effects models with time (day 1, 7 and 28) and treatment group (standard feed, inulin or cowpea) as fixed effects. Post hoc multiple comparisons were done via Tukey's test.

Survival analyses were performed in R using the 'survival' and 'survminer' packages. For 28-day mortality, Kaplan-Meier plots were constructed according to treatment group, statistical difference between intervention arms was assessed via the log-rank test. To assess the association between baseline PYY and 28-day mortality, Kaplan-Meier plots were first constructed according to PYY quartile (to allow for graphical representation of the effect), with statistical difference between the quartiles assessed via the log-rank test. Cox proportional hazard models were then constructed to adjust for potentially confounding variables. PYY was entered as a continuous variable into these models. The proportional hazards assumption was verified using Schoenfield residuals.

The SCFA from GCMS were analyzed using Agilent Mass Hunter Workstation Software for Quantitative Analysis. Peaks were inspected and corrected manually according to their retention times, including for IS. The area under the curve was obtained and normalized with the respective intensity of the IS and calibration curves used. Results were expressed in concentration levels with μM as the unit of measurement.

Processing of spectral data was conducted using Topspin v3.1 (Bruker, Karlsruhe Germany). Both SIMCA-P+ (Umetrics, Umea Sweden) and MATLAB (The MathWorks Inc., Natick USA) were used to generate unsupervised (PCA: Principal Component Analysis) and supervised (PLS: Projection to Latent Structures and O-PLS: Orthogonal Projection to Latent Structures) discriminant and regression models and associated scores and loadings plots. Metabolite identification used statistical correlation spectroscopy (STOCSY)[68] and subset optimization by reference matching (STORM)[69] to retrieve chemical shift patterns. Peak multiplicities were determined using J-resolved spectra. These data were then compared with an in-house database and the Human Metabolome Database[70] to identify metabolites.

16S rRNA data was processed as follows using the Mothur pipeline (Kozich et al., 2013) based on the online SOP accessed 14th July 2017. Paired read sequences were combined to maximize the information available for phylogenetic classification, and those with ambiguous bases or of the wrong length excluded. Similar paired sequences were clustered together as operational taxonomic units (OTUs), and classified by comparison to reference databases including SILVA,[71] Greengenes,[72] and the Ribosomal Database Project.[73] Median read depth was 25141, ranging from 13724 to 36142. Data were subsampled to 13724 reads as the minimum

number of reads observed in the sample set, which resulted in Good's coverage estimate of 99.9% (mean) 0.03% (SD), 99.9% (median). OTU data generated from the Mothur pipeline with corresponding taxonomy information was analyzed in the following ways. Univariate comparisons based on associated metadata were undertaken in STAMP software (statistical analysis of taxonomic and functional profiles) (Parks et al., 2014). Two group comparisons were conducted using two-sided White's non-parametric t test, with Benjamini-Hochberg false discovery rate (FDR) (Benjamini and Hochberg, 1995) applied to correct for multiple testing. Multiple group comparisons were conducted by the Kruskal-Wallis H-test, also using Benjamini-Hochberg FDR correction, with pairwise comparison made by post hoc Tukey-Kramer test. For clarity, corrected p values which may be greater than 1.000 following multiple testing correction have been capped at $p = 1.000$. Mothur scripts were used to calculate richness (Chao1 and Abundance-based Coverage Estimator (ACE)) and alpha diversity statistics (Inverse Simpson Index and Inverse Shannon Index), which were then compared using general statistical methods described above.

ADDITIONAL RESOURCES

Clinical trial registry number: PACTR201805003381361 http://www.pactr.org

Cell Reports Medicine, Volume *2*

Supplemental information

Modifying gut integrity and microbiome in children with severe acute malnutrition using legume-based feeds (MIMBLE): A pilot trial

Nuala Calder, Kevin Walsh, Peter Olupot-Olupot, Tonny Ssenyondo, Rita Muhindo, Ayub Mpoya, Jerusa Brignardello, Xuedan Wang, Eleanor McKay, Douglas Morrison, Elaine Holmes, Gary Frost, and Kathryn Maitland

Supplementary Tables 1: Baseline Characteristics

	ConF	InF	CpF	Total
Participants, n	18	20	20	58
Median age in months [Interquartile range)	21 [13-30]	18 [13-23]	18 [14-22]	18 [13-23]
Sex: Male	7 (39)	12 (60)	8 (40)	27 (47)
**Nutritional status and history**				
Median mid-upper arm circumference, cm [IQR]	11.1 [10.4-12.7]	10.5 [10.0-12.0]	11.0 [10.2-11.7]	10.9 [10.2-12.2]
Weight-for-height/length z score <3	10 (56)	13 (65)	12 (60)	35 (60)
Oedema (kwashiorkor)	11 (61)	13 (65)	13 (65)	37 (64)
Oedema severity: severe/generalized	8/11 (72)	10/13 (76)	13/13 (100)	31/37 (84)
Age when feeds introduced (month)	5 [2-6]	3.5 [2.8-6]	5 [2-6]	5 [2-6]
Breast feeding	4/17 (24)	3 (15)	5/19 (26)	12/56 (21)
Previous admission with SAM	1/17 (6)	2/19 (11)	1/19 (5)	4/55 (7)
**Complications at Presentation**				
Diarrhoea	2 (11)	2 (10)	9/19 (47)	13/57 (23)
Pneumonia or Pulmonary Tuberculosis	6 (33)	3 (15)	6/19 (32)	15/57 (26)
Malaria film positive	2/16 (13)	2/17 (12)	0/14 (0)	4/47 (8.5)
HIV Antibody positive	1/15 (7)	1/17 (6)	1/16 (6)	3/48 (6.3)
**Clinical Parameters at Presentation**				
Fever > 37.5^{o}C	4 (22)	8 (40)	6/19 (32)	18/57 (32)
Hypothermia (<36.0 ^{o}C)	1 (6)	1 (5)	1/19 (5)	3/57 (5)
Indrawing or deep breathing	1 (6)	2 (10)	3/19 (16)	6/57 (11)
Signs of severe dehydration	0 (0)	1 (5)	6/19 (32)	7/57 (12)
Hyponatraemia (<130 mmol/L)	4/15 (27)	10/18 (55)	11/17 (64)	25/50 (50)
Hypokalaemia (<3.0 mmol/L)	3/16 (19)	11/18 (61)	5/18 (28)	19/52 (37)
Hypoglycaemia (< 3mmol/dl)	2/17 (12)	0/19 (0)	2/18 (11)	4/54 (7)
Severe anaemia (Hb < 5g.dl)	2 (11)	2 (10)	1/19 (5)	5/57 (9)
Lactate > 2 mmols/L	14/17 (82)	15 (75)	10/19 (56)	39/56 (70)
**Risk Score***				
PET score (mean±SD)	1.00 (0.84)	0.95 (0.83)	1.16 (0.69)	1.03 (0.77)

Data are number (%) or median [interquartile range] unless otherwise specified.
% any of capillary refilling time > 2 seconds, temperature gradient or weak pulse
*Risk score: based Paediatric Emergency Triage Score (PET) (George et al 2015)
ConF: Standard recovery feeds F75/F100
InF: Standard recovery feeds with added standard volume of inulin as positive fermentable carbohydrate control
CpF: Standard recovery feeds enriched with cowpea flour
Supplemental Table 1 relates to Figure 1

Supplemental Table 2 Cox proportional hazards model for time to death (up to 28 days)

Variable	Adjusted Hazard Ratio	Lower 95% CI	Upper 95% CI	P
Age (per month)	1.05	0.89	1.02	0.14
Sex (Male vs Female)	1.71	0.47	6.2	0.41
InF versus ConF	1.63	0.33	8.1	0.54
CpF versus ConF	0.66	0.12	3.5	0.63
Paediatric Emergency Triage Score	1.53	0.61	3.8	0.36
Day 1 PYY (per st dev change)	2.8	1.6	5.1	0.0004

Supplemental Table 2 relates to Figure 2

Supplemental Table 3 Summary of biochemical indices and gut microbial phyla arranged by intervention arm and time point, with comparison between time points within intervention groups

	Control Standard Feeds (ConF)				Inulin-enriched Feeds (InF)				Cowpea-enriched Feeds (CpF)			
	ConF1 (n=16)	ConF7 (n=13)	ConF28 (n=13)	Within Group Comparison†	InF1 (n=19)	InF7 (n=13)	InF28 (n=10)	Within Group Comparison†	CpF1 (n=18)	CpF7 (n=15)	CpF28 (n=15)	Within Group Comparison†
Gut hormones (mean (SD))												
PYY pre-feed (pmol/L)	80.5 (93.9)	50.1 (48.6)	25.4 (11.1)	p=0.066	102.1 (83.6)^{InF28}	58.3 (58.8)	26.7 (19.2)^{InF1}	p=0.011	91.8 (74.8)^{CpF28}	55.9 (47.8)	33.8 (16.7)^{CpF1}	p=0.014
PYY post-feed (pmol/L)	82.9 (103.9)	58.4 (72.1)	31.4 (26.7)	p=0.197	98.4 (89.1)^{InF28}	60.2 (68.4)	29.9 (15.0)^{InF1}	p=0.045	65.0 (52.8)	46.7 (34.3)	37.7 (28.0)	p=0.164
GLP-1 pre-feed (pmol/L)	0.8 (0.8)	0.8 (0.3)	1.0 (0.8)	p=0.644	0.7 (0.4)	0.6 (0.4)	0.8 (0.5)	p=0.695	0.8 (0.5)	0.9 (0.7)	1.2 (0.8)	p=0.209
GLP-1 post feed (pmol/L)	0.5 (0.3)$^{ConF7, ConF28}$	0.8 (0.3)^{ConF1}	0.9 (0.3)^{ConF1}	p=0.006	0.8 (0.5)	0.8 (0.5)	0.9 (0.6)	p=0.887	0.8 (0.4)	0.9 (0.5)	1.0 (0.7)	p=0.701
Faecal Short Chain Fatty Acids (mean (SD))												
Acetate (µmol/L)	1651.0 (770.4)	1091.5 (604.4)^{ConF28}	1853.2 (829.7)^{ConF7}	p=0.021	1413.3 (475.0)	1086.5 (519.0)^{InF28}	1705.4 (673.4)^{InF7}	p=0.035	1542.5 (720.7)	1354.7 (766.8)	1782.4 (768.9)	p=0.287
Butyrate (µmol/L)	843.8 (877.3)^{ConF7}	241.7 (407.3)$^{ConF1, ConF28}$	1285.3 (816.1)^{ConF7}	p=0.004	753.7 (805.1)^{InF28}	361.5 (538.0)^{InF28}	1470.0 (1102.9)$^{InF1, InF7}$	p=0.009	760.5 (759.7)	649.4 (638.7)	706.7 (797.7)	p=0.858
Propionate (µmol/L)	1183.3 (1188.8)^{ConF7}	508.1 (419.9)$^{ConF1, ConF28}$	1697.8 (867.5)^{ConF7}	p=0.003	721.7 (695.8)	517.1 (840.8)	958.2 (736.2)	p=0.375	1229.0 (980.5)	1297.0 (1135.8)	1228.6 (544.1)	p=0.978
Dual Sugar Test (Median (IQR))												
Lactulose (% recovered)	0.22 (0.24)	0.94 (0.99)	0.41 (0.53)	p=0.089	0.30 (0.15)	0.38 (0.55)	0.72 (0.28)	p=0.527	0.13 (0.21)^{CpF28}	0.19 (0.06)	0.36 (0.53)^{CpF1}	p=0.017
Mannitol (% recovered)	1.05 (0.50)	3.15 (5.01)	2.71 (4.98)	p=0.530	0.92 (0.77)^{InF28}	2.27 (0.94)	6.70 (1.93)^{InF1}	p=0.012	0.16 (0.67)^{CpF28}	1.02 (1.77)	2.64 (3.06)^{CpF1}	p=0.038
L:M	0.21 (0.29)	0.36 (0.63)	0.14 (0.10)	p=0.370	0.26 (1.38)	0.35 (0.28)	0.11 (0.18)	p=0.403	0.16 (2.50)	0.17 (0.23)	0.23 (0.16)	p=0.961
Gut microbial species richness and diversity (mean (SD))												
Chao1	53.2 (24.0)	40.8 (12.9)	56.2 (17.0)	p=0.101	54.2 (31.2)^{InF7}	31.1 (13.2)^{InF1}	45.0 (19.7)	p=0.040	54.8 (32.4)	41.6 (21.7)	45.8 (14.8)	p=0.301
Abundance-based Coverage Estimator (ACE)	55.5 (20.9)	42.2 (13.4)^{ConF28}	61.3 (17.5)^{ConF7}	p=0.027	57.2 (32.3)	35.4 (12.7)	61.2 (37.2)	p=0.067	56.0 (31.0)	47.9 (20.6)	57.2 (31.2)	p=0.613
Inverse Simpson Index	4.7 (2.9)	3.2 (1.5)^{ConF28}	6.1 (2.1)^{ConF7}	p=0.011	4.6 (4.0)	2.4 (0.7)	4.6 (1.7)	p=0.084	4.4 (2.3)^{CpF7}	2.8 (1.2)$^{CpF1, CpF28}$	4.2 (1.6)^{CpF7}	p=0.002
Inverse Shannon Index	1.8 (0.7)	1.4 (0.5)^{ConF28}	2.2 (0.4)^{ConF7}	p=0.006	1.7 (0.7)^{InF7}	1.2 (0.4)$^{InF1, InF28}$	1.9 (0.5)^{InF7}	p=0.013	1.8 (0.6)	1.3 (0.5)	2.0 (0.4)	p=0.003
Gut Microbial Phyla Relative Abundances (%, mean (SD))*												
Actinobacteria	12.9 (13.1)	15.5 (16)	11.2 (10.6)	BH-FDR p=1.000	11.8 (11.3)	8.6 (9.2)	7.2 (5.3)	BH-FDR p=0.632	11.4 (10.4)	7.2 (8.9)	14.1 (10.8)	BH-FDR p=0.311
Bacteria unclassified	0.2 (0.4)	0 (0)	0 (0)	BH-FDR p=0.731	0.5 (1.5)	0 (0.1)	0.5 (0.8)	BH-FDR p=0.729	0.2 (0.3)	0.1 (0.3)	0.1 (0.1)	BH-FDR p=0.938
Bacteroidetes	16.1 (18.7)	7.8 (5.6)	21.3 (15.8)	BH-FDR p=0.226	8.5 (9.7)	5 (6.7)	23.6 (22.1)	BH-FDR p=0.573	8.1 (9.9)	4.3 (5.4)	9.1 (10.5)	BH-FDR p=0.772

Candidatus Saccharibacteria	0 (0)	0 (0)	0 (0)	BH-FDR p=0.666	0 (0)	0 (0)	0 (0)	BH-FDR p=0.798	0 (0)	0 (0)	0 (0)	BH-FDR p=0.866
Deferribacteres	0 (0)	0 (0)	0 (0)	-	0 (0)	0 (0)	0 (0)	-	0 (0)	0 (0)	0 (0)	BH-FDR p=0.807
Elusimicrobia	0 (0)	0 (0)	0 (0)	-	0 (0)	0 (0)	0 (0.1)	BH-FDR p=0.437	0 (0)	0 (0)	0 (0)	BH-FDR p=1.000
Firmicutes	31.6 (16)	26.9 (16.3)[ConF28]	47.7 (15.1)[ConF7]	BH-FDR p=0.038	32.3 (16.8)	25.1 (8.6)	37.4 (15.2)	BH-FDR p=0.503	41.3 (20.2)	31.7 (13.3)	49.9 (15)	BH-FDR p=0.117
Fusobacteria	0.4 (0.7)	0 (0.2)	1.3 (3.9)	BH-FDR p=0.264	0.3 (1.1)	0 (0)	4.1 (12.2)	BH-FDR p=0.418	1.3 (3.1)	0 (0.1)	0.1 (0.2)	BH-FDR p=0.861
Proteobacteria	38.2 (24.3)	49.7 (24.1)	17.8 (14)	BH-FDR p=0.053	45.7 (20)	61.2 (9.5)[InF28]	27.2 (22.6)[InF7]	BH-FDR p=0.029	37.3 (20.5)	56.3 (17.4)[CpF28]	25.4 (18.3)[CpF7]	BH-FDR p=0.013
Spirochaetes	0 (0)	0 (0)	0 (0)	BH-FDR p=0.824	0.6 (2.4)	0 (0)	0 (0)	BH-FDR p=0.537	0.5 (1.8)	0 (0.1)	0 (0)	BH-FDR p=0.900
Synergistetes	0 (0)	0 (0)	0 (0)	BH-FDR p=0.721	0 (0)	0 (0)	0 (0)	-	0 (0)	0 (0)	0 (0)	-
Tenericutes	0 (0)	0 (0)	0 (0)	BH-FDR p=0.548	0 (0)	0 (0)	0 (0)	BH-FDR p=0.644	0 (0)	0 (0)	0 (0)	BH-FDR p=0.989
Verrucomicrobia	0.7 (2.1)	0.1 (0.2)	0.6 (1.6)	BH-FDR p=0.194	0.4 (1.7)	0 (0)	0 (0.1)	BH-FDR p=0.790	0 (0)	0.4 (1.4)	1.3 (4.5)	BH-FDR p=0.986
Bifidobacterium Genus Relative Abundance (%, mean (SD))												
Bifidobacterium	10.8 (12.5)	13.6 (14.0)	8.9 (10.3)	BH-FDR p=0.894	10.4 (11.4)	8.3 (9.3)	5.7 (5.8)	BH-FDR p=0.850	8.2 (8.2)	5.8 (8.6)	10.9 (8.7)	BH-FDR p=1.000

Abbreviations: PYY: peptide tyrosine tyrosine; GLP-1: glucagon-like peptide 1; L:M: lactulose:mannitol ratio; SD: standard deviation; IQR: interquartile range; BH-FDR p: Benjamini-Hochberg False Discovery Rate Corrected p-value

†Between Group Comparisons report one-way ANOVA (or Kruskal-Wallis one-way ANOVA if non-parametric) p-value with significant ($p<0.05$) pairwise comparisons indicated by superscript determined by Tukey's post-hoc test (or Dunn's post hoc test if Kruskal-Wallis used).

16S rRNA relative abundances compared using multiple groups by Kruskal-Wallis H Test, pairwise Post-Hoc Tukey-Kramer and p-values multiple test correction by Benjamini-Hochberg False Discovery Rate and significant (corrected $p<0.05$) pairwise comparisons indicated by superscript determined by post-hoc Tukey-Kramer

Gut microbial phyla determined by 16S rRNA sequencing; *those with 0% relative abundance and SD were not detected in the 16S rRNA sequencing

[ConF1, ConF7, ConF28] denotes post hoc pairwise significant difference from Control Standard Feed Day 1, Control Standard Feed Day 7, Control Standard Feed Day 28 respectively

[InF1, InF7, InF28] denotes post hoc pairwise significant difference from Inulin-enriched Feed Day 1, Inulin-enriched Feed Day 7, Inulin-enriched Feed Day 28 respectively

[CpF1, CpF7, CpF28] denotes post hoc pairwise significant difference from Cowpea-enriched Feed Day 1, Cowpea-enriched Feed Day 7, Cowpea-enriched Feed Day 28 respectively

Supplemental Table 3 relates to Figure 4

Supplemental Table 4: Summary of biochemical indices and gut microbial phyla arranged by intervention arm and time point, with comparison between intervention groups at each study time point

	Day 1				Day 7				Day 28			
	ConF1 (n=16)	InF1 (n=19)	CpF1 (n=18)	Between Group Comparison‡	ConF7 (n=13)	InF7 (n=13)	CpF7 (n=15)	Between Group Comparison‡	ConF28 (n=13)	InF28 (n=10)	CpF28 (n=15)	Between Group Comparison‡
Gut hormones (mean (SD))												
PYY pre-feed (pmol/L)	80.5 (93.9)	102.1 (83.6)	91.8 (74.8)	p=0.741	50.1 (48.6)	58.3 (58.8)	55.9 (47.8)	p=0.908	25.4 (11.1)	26.7 (19.2)	33.8 (16.7)	p=0.338
PYY post-feed (pmol/L)	82.9 (103.9)	98.4 (89.1)	65.0 (52.8)	p=0.490	58.4 (72.1)	60.2 (68.4)	46.7 (34.3)	p=0.798	31.4 (26.7)	29.9 (15.0)	37.7 (28.0)	p=0.693
GLP-1 pre-feed (pmol/L)	0.8 (0.8)	0.7 (0.4)	0.8 (0.5)	p=0.908	0.8 (0.3)	0.6 (0.4)	0.9 (0.7)	p=0.436	1.0 (0.8)	0.8 (0.5)	1.2 (0.8)	p=0.406
GLP-1 post feed (pmol/L)	0.5 (0.3)[CpF1]	0.8 (0.5)	0.8 (0.4)[ConF1]	p=0.041	0.8 (0.3)	0.8 (0.5)	0.9 (0.5)	p=0.724	0.9 (0.3)	0.9 (0.6)	1.0 (0.7)	p=0.851
Faecal Short Chain Fatty Acids (mean (SD))												
Acetate (µmol/L)	1651.0 (770.4)	1413.3 (475.0)	1542.5 (720.7)	p=0.167	1091.5 (604.4)	1086.5 (519.0)	1354.7 (766.8)	p=0.455	1853.2 (829.7)	1705.4 (673.4)	1782.4 (768.9)	p=0.901
Butyrate (µmol/L)	843.8 (877.3)	753.7 (805.1)	760.5 (759.7)	p=0.339	241.7 (407.3)	361.5 (538.0)	649.4 (638.7)	p=0.135	1285.3 (816.1)	1470.0 (1102.9)	706.7 (797.7)	p=0.089
Propionate (µmol/L)	1183.3 (1188.8)	721.7 (695.8)	1229.0 (980.5)	p=0.187	508.1 (419.9)	517.1 (840.8)	1297.0 (1135.8)	p=0.029	1697.8 (867.5)	958.2 (736.2)	1228.6 (544.1)	p=0.053
Dual Sugar Test (Median (IQR))												
Lactulose (% recovered)	0.22 (0.24)	0.30 (0.15)	0.13 (0.21)	p=0.241	0.94 (0.99)[CpF7]	0.38 (0.55)	0.19 (0.06)[ConF7]	p=0.017	0.41 (0.53)	0.72 (0.28)	0.36 (0.53)	p=0.712
Mannitol (% recovered)	1.05 (0.50)	0.92 (0.77)	0.16 (0.67)	p=0.194	3.15 (5.01)	2.27 (0.94)	1.02 (1.77)	p=0.311	2.71 (4.98)	6.70 (1.93)	2.64 (3.06)	p=0.158
L:M	0.21 (0.29)	0.26 (1.38)	0.16 (2.50)	p=0.754	0.36 (0.63)	0.35 (0.28)	0.17 (0.23)	p=0.465	0.14 (0.10)	0.11 (0.18)	0.23 (0.16)	p=0.634
Gut microbial species richness and diversity (mean (SD))												
Chao1	53.2 (24.0)	54.2 (31.2)	54.8 (32.4)	p=0.987	40.8 (12.9)	31.1 (13.2)	41.6 (21.7)	p=0.206	56.2 (17.0)	45.0 (19.7)	45.8 (14.8)	p=0.197
Abundance-based Coverage Estimator (ACE)	55.5 (20.9)	57.2 (32.3)	56.0 (31.0)	p=0.984	42.2 (13.4)	35.4 (12.7)	47.9 (20.6)	p=0.142	61.3 (17.5)	61.2 (37.2)	57.2 (31.2)	p=0.917
Inverse Simpson Index	4.7 (2.9)	4.6 (4.0)	4.4 (2.3)	p=0.969	3.2 (1.5)	2.4 (0.7)	2.8 (1.2)	p=0.255	6.1 (2.1)	4.6 (1.7)	4.2 (1.6)	p=0.162
Inverse Shannon Index	1.8 (0.7)	1.7 (0.7)	1.8 (0.6)	p=0.959	1.4 (0.5)	1.2 (0.4)	1.3 (0.5)	p=0.494	2.2 (0.4)	1.9 (0.5)	2.0 (0.4)	p=0.202
Gut Microbial Phyla Relative Abundances (%, mean (SD))*												
Actinobacteria	12.9 (13.1)	11.8 (11.3)	11.4 (10.4)	BH-FDR p=0.953	15.5 (16)	8.6 (9.2)	7.2 (8.9)	BH-FDR p=0.988	11.2 (10.6)	7.2 (5.3)	14.1 (10.8)	BH-FDR p=0.631
Bacteria unclassified	0.2 (0.4)	0.5 (1.5)	0.2 (0.3)	BH-FDR p=0.839	0 (0)	0 (0.1)	0.1 (0.3)	BH-FDR p=0.954	0 (0)	0.5 (0.8)	0.1 (0.1)	BH-FDR p=0.701
Bacteroidetes	16.1 (18.7)	8.5 (9.7)	8.1 (9.9)	BH-FDR p=1.000	7.8 (5.6)	5 (6.7)	4.3 (5.4)	BH-FDR p=1.000	21.3 (15.8)	23.6 (22.1)	9.1 (10.5)	BH-FDR p=1.000

Candidatus Saccharibacteria	0 (0)	0 (0)	0 (0)	BH-FDR p=0.838	0 (0)	0 (0)	0 (0)	BH-FDR p=0.983	0 (0)	0 (0)	0 (0)	BH-FDR p=1.000
Deferribacteres	0 (0)	0 (0)	0 (0)	-	0 (0)	0 (0)	0 (0)	-	0 (0)	0 (0)	0 (0)	-
Elusimicrobia	0 (0)	0 (0)	0 (0)	BH-FDR p=0.983	0 (0)	0 (0)	0 (0)	BH-FDR p=1.000	0 (0)	0 (0.1)	0 (0)	BH-FDR p=0.641
Firmicutes	31.6 (16)	32.3 (16.8)	41.3 (20.2)	BH-FDR p=0.863	26.9 (16.3)	25.1 (8.6)	31.7 (13.3)	BH-FDR p=1.000	47.7 (15.1)	37.4 (15.2)	49.9 (15)	BH-FDR p=0.562
Fusobacteria	0.4 (0.7)	0.3 (1.1)	1.3 (3.1)	BH-FDR p=1.000	0 (0.2)	0 (0)	0 (0.1)	BH-FDR p=1.000	1.3 (3.9)	4.1 (12.2)	0.1 (0.2)	BH-FDR p=0.417
Proteobacteria	38.2 (24.3)	45.7 (20)	37.3 (20.5)	BH-FDR p=0.858	49.7 (24.1)	61.2 (9.5)	56.3 (17.4)	BH-FDR p=0.873	17.8 (14)	27.2 (22.6)	25.4 (18.3)	BH-FDR p=0.821
Spirochaetes	0 (0)	0.6 (2.4)	0.5 (1.8)	BH-FDR p=0.990	0 (0)	0 (0)	0 (0.1)	BH-FDR p=1.000	0 (0)	0 (0)	0 (0)	-
Synergistetes	0 (0)	0 (0)	0 (0)	-	0 (0)	0 (0)	0 (0)	-	0 (0)	0 (0)	0 (0)	-
Tenericutes	0 (0)	0 (0)	0 (0)	BH-FDR p=1.000	0 (0)	0 (0)	0 (0)	BH-FDR p=0.733	0 (0)	0 (0)	0 (0)	BH-FDR p=0.560
Verrucomicrobia	0.7 (2.1)	0.4 (1.7)	0 (0)	BH-FDR p=0.023	0.1 (0.2)	0 (0)	0.4 (1.4)	BH-FDR p=1.000	0.6 (1.6)	0 (0.1)	1.3 (4.5)	BH-FDR p=0.553
Bifidobacterium Genus Relative Abundance (%, mean (SD))												
Bifidobacterium	10.8 (12.5)	10.4 (11.4)	8.2 (8.2)	BH-FDR p=1.000	13.6 (14.0)	8.3 (9.3)	5.8 (8.6)	BH-FDR p=1.000	8.9 (10.3)	5.7 (5.8)	10.9 (8.7)	BH-FDR p=1.000

Abbreviations: PYY: peptide tyrosine tyrosine; GLP-1: glucagon-like peptide 1; L:M: lactulose:mannitol ratio; SD: standard deviation; IQR: interquartile range; BH-FDR p: Benjamini-Hochberg False Discovery Rate Corrected p-value

†Between Group Comparisons report one-way ANOVA (or Kruskal-Wallis one-way ANOVA if non-parametric) p-value with significant ($p<0.05$) pairwise comparisons indicated by superscript determined by Tukey's post-hoc test (or Dunn's post hoc test if Kruskal-Wallis used).

16S rRNA relative abundances compared using multiple groups by Kruskal-Wallis H Test, pairwise Post-Hoc Tukey-Kramer and p-values multiple test correction by Benjamini-Hochberg False Discovery Rate and significant (corrected $p<0.05$) pairwise comparisons indicated by superscript determined by post-hoc Tukey-Kramer

Gut microbial phyla determined by 16S rRNA sequencing; *those with 0% relative abundance and SD were not detected in the 16S rRNA sequencing

$^{ConF1, ConF7, ConF28}$ denotes post hoc pairwise significant difference from Control Standard Feed Day 1, Control Standard Feed Day 7, Control Standard Feed Day 28 respectively

$^{InF1, InF7, InF28}$ denotes post hoc pairwise significant difference from Inulin-enriched Feed Day 1, Inulin-enriched Feed Day 7, Inulin-enriched Feed Day 28 respectively

$^{CpF1, CpF7, CpF28}$ denotes post hoc pairwise significant difference from Cowpea-enriched Feed Day 1, Cowpea-enriched Feed Day 7, Cowpea-enriched Feed Day 28 respectively

Supplemental Table 4 relates to Figure 4

Supplemental Table 5 Antibiotic treatments prescribed at any stage from admission to day 28 in each study arm

**Antibiotic, n(%)**	**ConF (n=18)**	**InF (n=20)**	**CpF (n=20)**	**Pearson X^2**	**p-value**
Amoxycillin	1 (5.6)	1 (5.0)	1 (5.0)	0.008*	0.996
Ampicillin	10 (55.6)	14 (70.0)	10 (50.0)	1.750	0.417
Ampiclox	1 (5.6)	3 (15.0)	1 (5.0)	1.493*	0.474
Benzylpenicillin	0 (0)	1 (5.0)	1 (5.0)	1.518*	0.468
Ceftriaxone	12 (66.7)	12 (60.0)	13 (65.0)	0.202	0.904
Chloramphenicol	0 (0)	1 (5.0)	1 (5.0)	1.518*	0.468
Ciprofloxacin	0 (0)	1 (5.0)	0 (0)	2.163*	0.339
Cloxacillin	0 (0)	2 (10.0)	2 (10.0)	3.104*	0.212
Erythromycin	0 (0)	1 (5.0)	0 (0)	2.163*	0.339
Ethambutol	0 (0)	0 (0)	1 (5.0)	2.163*	0.339
Gentamycin	13 (72.2)	17 (85.0)	14 (70.0)	1.496*	0.473
Isoniazid	0 (0)	0 (0)	1 (5.0)	2.163*	0.339
Metronidazole	0 (0)	1 (5.0)	0 (0)	2.163*	0.339
Pyrazinamide	0 (0)	0 (0)	1 (5.0)	2.163*	0.339
Rifampin	0 (0)	0 (0)	1 (5.0)	2.163*	0.339
Cotrimoxazole	0 (0)	0 (0)	1 (5.0)	2.163*	0.339
Number of Different Antibiotics Prescribed					
None	1 (5.6)	2 (10.0)	4 (20.0)	1.979	0.372
One only	3 (16.8)	0 (0)	1 (5.0)	4.950*	0.084
Two	8 (44.4)	5 (25.0)	7 (35.0)	1.589	0.452
Three	6 (33.3)	8 (40.0)	3 (15.0)	3.220	0.200
Four	0 (0)	5 (25.0)	4 (20.0)	**7.553***	**0.023**
*Where the assumptions of the Pearson X^2 are violated, Likelihood Ratio statistic and associated p-value are presented instead.					

Supplemental Table 5 relates to STAR methods Clinical Monitoring section.

Supplemental Figure 1: Study flow diagram

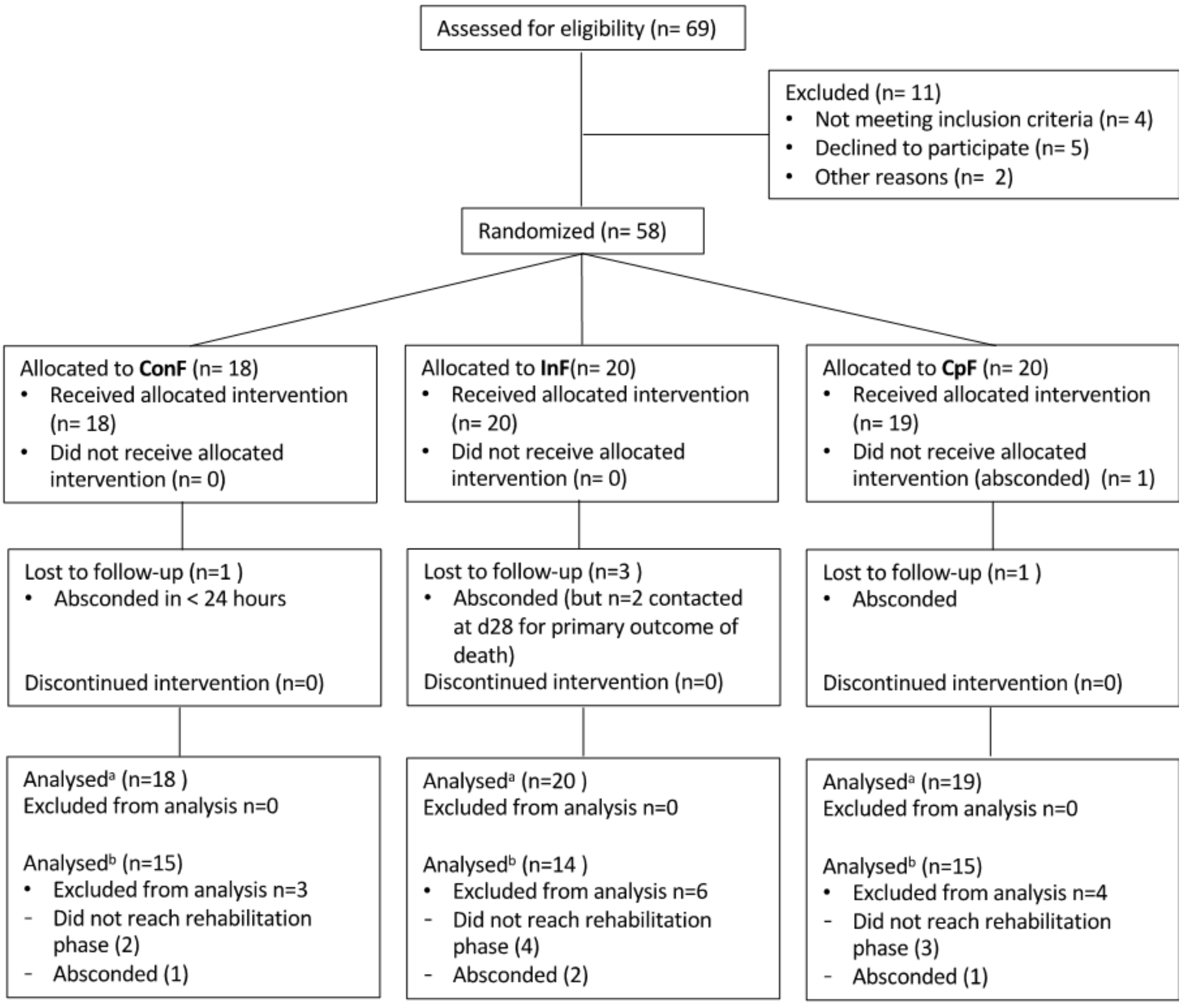

[a] *Analysed with respect to primary outcome survival up to 28 days (time to event analysis)*
[b] *Analysed with respect to primary outcome weight gain > 5g/kg/day (Participants who reached rehabilitation stage)*

Supplemental Figure 1 relates to STAR Methods Experimental Model and Subject Details
